# *Porphyromonas gingivalis* aggravates atherosclerotic plaque instability by promoting lipid-laden macrophage necroptosis

**DOI:** 10.1038/s41392-025-02251-6

**Published:** 2025-05-23

**Authors:** Xiaofei Huang, Mengru Xie, Yixuan Wang, Xiaofeng Lu, Feng Mei, Kaiwen Zhang, Xinlong Yang, Guangjin Chen, Ying Yin, Guangxia Feng, Wencheng Song, Nianguo Dong, Xuliang Deng, Songling Wang, Lili Chen

**Affiliations:** 1 Department of Stomatology, Union Hospital, Tongji Medical College, Huazhong University of Science and Technology, Wuhan, China; 2 School of Stomatology, Tongji Medical College, Huazhong University of Science and Technology, Wuhan, China; 3 Hubei Province Key Laboratory of Oral and Maxillofacial Development and Regeneration, Wuhan, China; 4 Department of Cardiovascular Surgery, Union Hospital, Tongji Medical College, Huazhong University of Science and Technology, Wuhan, China; 5 Key Laboratory of Organ Transplantation, Ministry of Education, NHC Key Laboratory of Organ Transplantation, Key Laboratory of Organ Transplantation, Chinese Academy of Medical Sciences, Wuhan, China; 6 Department of Geriatric Dentistry, NMPA Key Laboratory for Dental Materials, National Engineering Laboratory for Digital and Material, Technology of Stomatology, Beijing Laboratory of Biomedical Materials, Peking University School and Hospital of Stomatology, Beijing, China; 7 Laboratory of Homeostatic Medicine, School of Medicine, Southern University of Science and Technology, Shenzhen, China; 8 Beijing Laboratory of Oral Health, Capital Medical University, Beijing, China

**Keywords:** Infectious diseases, Cardiovascular diseases

## Abstract

At advanced phases of atherosclerosis, the rupture and thrombogenesis of vulnerable plaques emerge as primary triggers for acute cardiovascular events and fatalities. Pathogenic infection such as periodontitis-associated *Porphyromonas gingivalis* (*Pg*) has been suspected of increasing the risks of atherosclerotic cardiovascular disease, but its relationship with atherosclerotic plaque destabilization remains elusive. Here we demonstrated that the level of *Pg*-positive clusters positively correlated with the ratio of necrotic core area to total atherosclerotic plaque area in human clinical samples, which indicates plaque instability. In rabbits and *Apoe*^−/−^ mice, *Pg* promoted atherosclerotic plaque necrosis and aggravated plaque instability by triggering oxidative stress, which led to macrophage necroptosis. This process was accompanied by the decreased protein level of forkhead box O3 (FOXO3) in macrophages. The mechanistic dissection showed that *Pg* lipopolysaccharide (LPS) evoked macrophage oxidative stress via the TLR4 signaling pathway, which subsequently activated MAPK/ERK-mediated FOXO3 phosphorylation and following degradation. While the gingipains, a class of proteases produced by *Pg*, could effectively hydrolyze FOXO3 in the cytoplasm of macrophages. Both of them decreased the nuclear level of FOXO3, followed by the release of histone deacetylase 2 (HDAC2) from the macrophage scavenger receptor 1 (*Msr1*) promoter, thus promoting *Msr1* transcription. This enhanced MSR1-mediated lipid uptake further amplified oxidative stress-induced necroptosis in lipid-laden macrophages. In summary, *Pg* exacerbates macrophage oxidative stress-dependent necroptosis, thus enlarges the atherosclerotic plaque necrotic core and ultimately promotes plaque destabilization.

## Introduction

Atherosclerotic cardiovascular diseases (ASCVDs)^1^ is account for ~1/3rd of fatalities among worldwide cardiovascular disease cases.^2^ Atherosclerosis, a vascular chronic inflammatory disorder triggered by lipid metabolic dysfunction, can ultimately develop into clinically overt ASCVDs. During the advanced stages of atherosclerosis, arterial plaques are gradually developing from stable to unstable, culminating in plaque rupture, detachment, intraplaque hemorrhage (IPH), and thrombus formation.^3^ These unstable plaques are not equivalent to severely obstructive plaques, more importantly, they may pose greater clinical risks due to their high thrombogenic potential.^4,5^ It is reported that these unstable plaques represent the primary etiological factors causing many major adverse cardiovascular events, including stroke,^6^ heart failure, and myocardial infarction (MI).^7^ Atherosclerotic plaque destabilization represents a chronic and multifactorial pathological progression. The incipient plaques are initiated by the subendothelial deposition of oxidized low-density lipoprotein (ox-LDL), and further expand under continuous lipid accumulation, chronic inflammation, and extracellular matrix remodeling, leading to arterial wall thickening and subsequent clinical ischemic heart disease. Under persistent pathophysiological stimuli, advanced plaques undergo structural and composition alterations characterized by progressive necrotic core expansion, weakening of the fibrous cap, collagen fiber degradation, as well as dense inflammatory cell infiltration.^8^ These phenomena collectively render the atherosclerotic plaques to destabilize. While the fundamental mechanisms driving atherosclerotic plaque formation and growth within the arterial wall have been extensively characterized, the pathological processes underlying the transition from benign stable lesions to unstable, rupture-prone plaques remain poorly understood.

Lipids and inflammation are recognized as canonical and predominant drivers exacerbating atherosclerotic plaque instability, yet important contributory factors continue to be uncovered.^9^ It is thought that chronic infectious diseases, including periodontal disease, facilitate ASCVDs development.^10,11^ Epidemiological evidences have revealed that patients with periodontitis exhibit a 1.27-fold higher prevalence of atherosclerosis compared with non-periodontitis individuals.^12^ Furthermore, periodontitis has been confirmed to be significantly linked with elevated risk of coronary heart disease, MI,^11^ stroke^13^ as well as peripheral arterial disease.^14^ *Porphyromonas gingivalis* (*Pg*), a primary pathogenic bacteria responsible for chronic periodontal disease etiology, is reported to be epidemiologically and mechanistically linked to atherosclerosis development.^15^ Pathological analyses confirm the presence of *Pg* within the thrombus specimens obtained from individuals with acute MI.^16,17^ Systemic exposure to *Pg* correlates with a heightened susceptibility to stroke in humans. Additionally, the levels of *Pg* and its plasma antibodies exhibited significant associations with abdominal aortic aneurysm diameter and thrombus volume in patients.^18^ According to extensive animal experimental studies, *Pg* infection through different ways, including oral administration, gavage, and intravenous injection, can all significantly promote the growth of atherosclerotic lesions in *Apoe*^*−/−*^ mice and potentially exacerbate plaque instability.^19,20^ *Pg* can colonize ischemic myocardial tissue in murine models of MI, promoting cardiac rupture and consequently increasing the mortality rates.^21^ This evidence underscores *Pg*’s possible contributory role in atherosclerotic plaque destabilization. However, phenotypic and mechanistic investigations about the association between *Pg* infection and plaque instability is still lacking.

Formation and expansion of the necrotic core serves as central determinants of plaque vulnerability. The intraplaque necrotic core is predominantly composed of cholesterol crystals, lipid, and cellular debris, but deficient in collagen matrix. The structurally vulnerable locus is prone to rupture, IPH and thrombosis.^22^ Histopathological analyses reveal that necrotic core burden exceeds 10% of total plaque area in 75% of thin fibrous cap atheroma, while surpassing 25% in 65% of ruptured plaques.^23^ The excessive demise of foam cells, mainly originated from macrophages and vascular smooth muscle cells (VSMCs) within the lesion, dominates the necrotic core progression. It is shown that the majority of macrophages and VSMCs in advanced human atheroma manifested necrotic ultrastructural features including cellular swelling, membrane rupture and organelle disintegration.^24^ Recent advances in the understanding of regulated necrosis have broadened our insights into cell death within atherosclerotic plaques. Common manners of regulated necrosis like pyroptosis, ferroptosis, and necroptosis are validated to be differentially activated during atherosclerosis progression and plaque destabilization.^22^ Unlike apoptosis, necrotic cells release immunostimulatory intracellular components, initiating sterile inflammatory cascades that exacerbate bystander cell death.^25^ These findings underscore necrosis as a principal contributor to necrotic core expansion and plaque vulnerability. Studies revealed that *Pg* can induce macrophage apoptosis,^26^ necroptosis,^27^ and pyroptosis,^28^ as well as VSMC apoptosis,^29^ but little is known about its contribution to necrotic core enlargement and the potential molecular mechanisms.

Here, we report that human atherosclerotic plaques enriched with higher levels of *Pg* are more unstable, and combined with studies in rabbits and mice, we demonstrate that *Pg* infection exacerbates lesion destabilization characterized by increased plaque necrosis through promoting oxidative stress-mediated necroptosis in lipid-laden macrophages. This oxidative stress not only originates from *Pg* stimulation, but is predominantly amplified by enhanced lipid uptake mediated by macrophage scavenger receptor 1 (MSR1). Mechanistically, *Pg*-derived virulence factors lipopolysaccharide (LPS) and gingipains upregulate MSR1 expression via disrupting the forkhead box O3 (FOXO3)-histone deacetylase 2 (HDAC2)-mediated transcriptional inhibition of *Msr1*, which promotes subsequent cascades leading to necroptotic cell death. Our findings indicate a causative link between periodontal pathogen infection and atherosclerotic plaque destabilization, and may offer novel perspectives into the therapeutic strategies targeting both disease prevention and clinical management against ASCVDs.

## Results

### *Pg* contributes to atherosclerotic plaque vulnerability

**Fig. 1:**
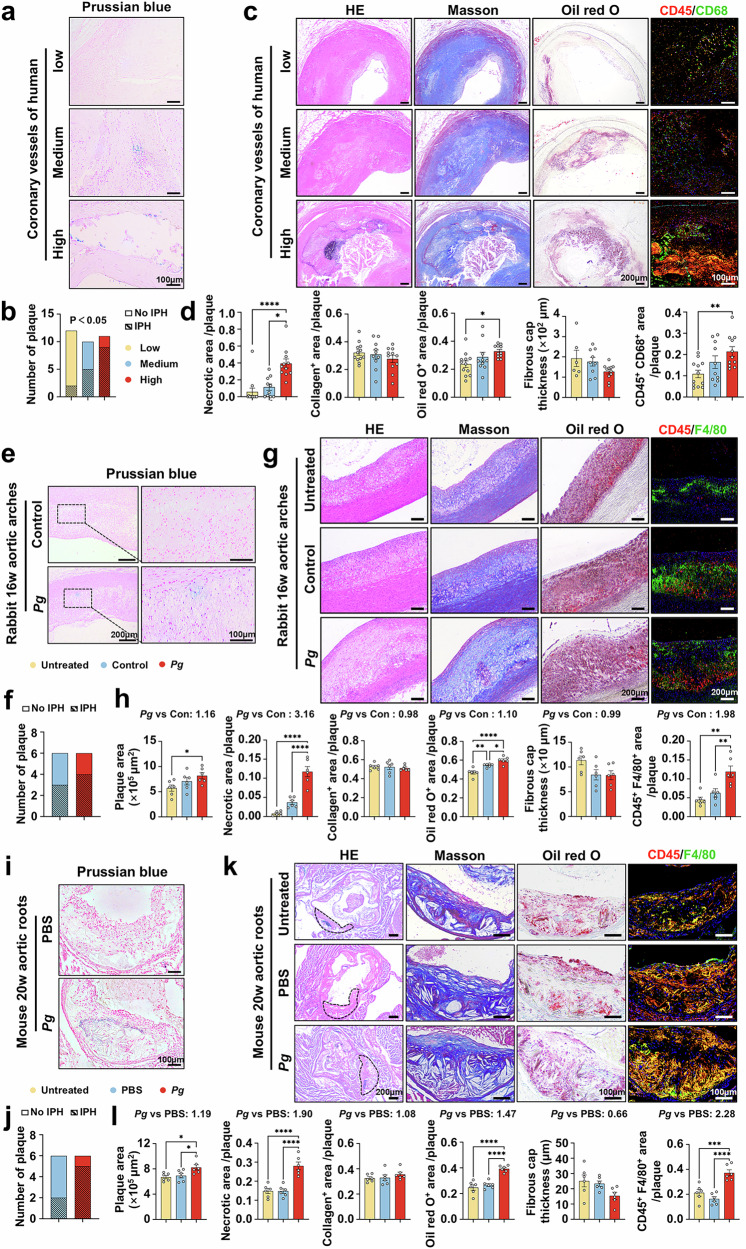
*Pg* contributes to atherosclerotic plaque vulnerability. **a** Intraplaque hemorrhage (IPH) detection with Prussian blue staining of human coronary atherosclerotic plaques. Scale bar = 100 μm. **b** Comparisons of the plaque numbers showing IPH in low, medium, and high groups of human coronary arteries. Group Low, *n* = 12; group medium, *n* = 10, group high, *n* = 11. **c** H&E, Masson, Oil Red O staining (scale bar = 200 μm), and CD45 and CD68 co-staining (scale bar = 100 μm) (macrophage marker) of human coronary plaques. Nuclei were labeled using DAPI. **d** Quantification of the ratio of necrotic area, collagen content, Oil Red O^+^ area, CD45 and CD68 positive areas of human coronary plaques, and the thickness of fibrous cap. Group low, *n* = 12; group medium, *n* = 10, group high, *n* = 11. Prussian blue staining of atherosclerotic plaques in aortic arches of rabbits with periodontal ligature and treated with or without *Pg* for 16 weeks (**e**) (left scale bar = 200 μm, right scale bar = 100 μm), and aortic roots of *Apoe*^−/−^ mice challenged with or without *Pg* for 20 weeks (**i**) (scale bar = 100 μm). Quantification of the plaque numbers showing IPH in aortic samples of rabbits (**f**), and *Apoe*^−/−^ mice (**j**). *n* = 6 per group. H&E, Masson, and Oil Red O staining, and CD45 and F4/80 co-staining (macrophage marker) of atherosclerotic plaques in aortic arches of rabbits (**g**) (scale bar = 200 μm), and aortic roots of *Apoe*^−/−^ mice (**k**) (scale bar = 200 μm in H&E, scale bar = 100 μm in the rest images). Nuclei were labeled using DAPI. Quantitative analyses of plaque size, necrotic area, collagen content, Oil Red O^+^ area, thickness of fibrous cap, CD45 and F4/80-positive areas of atherosclerotic plaques of rabbits (**h**), and *Apoe*^−/−^ mice (**l**). *n* = 6 per group. Results were represented as mean ± SEM. Data were analyzed by Fisher exact test (**b**, **f**, **j**), by Kruskal–Wallis test (necrotic area analysis in **d**), or by one-way ANOVA (**d**, **h**, **l**). ****P < 0.0001; ***P < 0.001; **P < 0.01; *P < 0.05

To investigate the influence of *Pg* infection on the vulnerability of atherosclerotic lesions, we initially collected atherosclerotic coronary plaques from 33 patients diagnosed with ASCVDs. Fluorescent in situ hybridization (FISH) was conducted using a FITC-labeled specific probe to detect and localize *Pg* within the plaques. Utilizing FISH, varying numbers of fluorescent clusters were observed within the plaques, confirming the localization of *Pg* (Supplementary Fig. 1a). Based on the number of *Pg* fluorescent clusters, the plaques were categorized into three groups: low (*Pg* clusters < 20), medium (20 ≤ *Pg* clusters < 80) and high (*Pg* clusters ≥ 80) groups. According to Virmani’s classification,^30^ the atherosclerotic plaque in our study were clarified into four categories, including pathological intimal thickening, fibrous cap atheroma/fibrocalcific plaque, thin fibrous cap atheroma, as well as plaque rupture. We found a strong positive association between the prevalence of *Pg*-clusters within plaques and plaque progression (Supplementary Fig. 1b). Compared to the plaques in medium and low groups, plaques in high groups exhibited a higher incidence of IPH, an important trigger of plaque vulnerability,^31^ through the Perls’ Prussian blue staining (Fig. 1a, b). Besides, we assessed the histological features of vulnerable plaques.^32^ The data revealed that plaques in high groups exhibited larger necrotic core, more lipid content, and macrophage accumulation relative to the plaques in medium and low groups, while the collagen^+^ area and fibrous cap thickness showed no difference (Fig. 1c, d). These thus indicate that plaques with higher levels of *Pg* present more vulnerable.

Next, we aimed to determine the contribution of *Pg* to atherosclerotic plaque vulnerability in rabbits and *Apoe*^−/−^ mice. Compared to rabbits without *Pg* inoculation, those infected with *Pg* presented more IPH (Fig. 1e, f), larger plaque size, bigger necrotic core, more lipid content, and greater macrophage infiltration in the aortic arches (Fig. 1g, h). Similarly, *Apoe*^−/−^ mice treated with *Pg* for 20 weeks exhibited more IPH (Fig. 1i, j), increased aortic root plaque area, necrotic area, lipid deposition, and macrophage infiltration compared to non-infected controls (Fig. 1k, l). These results delineate that *Pg* facilitates the atherosclerotic plaque destabilization.

The transition from early-stage plaques to vulnerable, advanced lesions evolves through a gradual and intricate process. Our study sought to delineate the mechanisms by which *Pg* aggravates plaque destabilization, commencing with an investigation of the early stages of atherosclerosis. Notably, *Apoe*^−/−^ mice inoculated with *Pg* for 14 weeks and 8 weeks also manifested a discernible rise in instability of the aortic plaques. This was characterized by a larger plaque size and necrotic area, more lipid accumulation, as well as a higher proportion of macrophages, while comparable levels of collagen and fibrous cap thickness were detected across the control and *Pg*-treated groups (Supplementary Figs. 2 and 3). Collectively, these data indicate a clear predisposition toward plaque instability in *Pg*-infected mice, spanning from early to late stages of atherosclerosis.

Furthermore, some studies have indicated that VSMCs, T cells, and neutrophils also played critical roles in plaque rupture.^33–35^ To comprehensively profile the aortic plaque cell composition, we analyzed the VSMC and immune cell enrichment across different groups. Human atherosclerotic plaques in high groups showed increased infiltration of CD45^+^CD68^+^ macrophages and CD3^+^ T cells, reduced content of VSMCs and no significant change in neutrophil levels (Fig. 1c, d and Supplementary Fig. 4a–c). Similarly, plaques from *Pg*-infected rabbits showed elevated aggregation of CD45^+^F4/80^+^ macrophages and CD3^+^ T cells, a decrease in VSMCs, and unchanged neutrophil levels (Fig. 1g, h and Supplementary Fig. 4d–f). Additionally, plaques in *Pg*-inoculated *Apoe*^−/−^ mice showed more accumulation of CD45^+^F4/80^+^ macrophages, while VSMCs, CD3^+^ T cells, and neutrophils remained unaltered (Fig. 1k, l and Supplementary Fig. 5).

### *Pg* infection induces lipid-laden macrophage necroptosis enlarging necrotic core

**Fig. 2:**
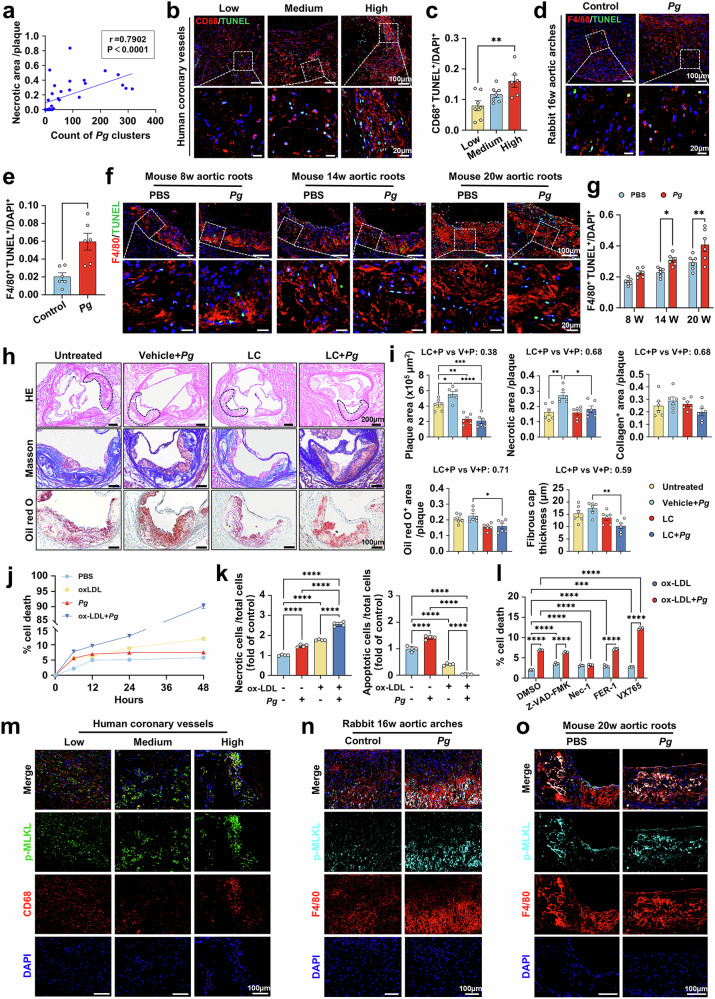
*Pg* infection induces lipid-laden macrophage necroptosis enlarging necrotic core. **a** Non-parametric Spearman’s test between the ratio of necrotic area and the amount of *Pg* positive clusters within the human coronary atherosclerotic plaques. *r* = 0.7902, *P* < 0.0001. *n* = 33. **b**–**g** Quantification of the proportion of dying macrophages probed by TUNEL and CD45-CD68/F4/80 co-staining in human coronary plaques (**b**, **c**) (group low, *n* = 7; group medium, *n* = 7, group high, *n* = 6), rabbit aortic arch plaques (**d**, **e**) (*n* = 6 per group) and mouse aortic root plaques (**f**, **g**) (*n* = 6 per group). Nuclei were labeled using DAPI. Scale bar = 100 μm on top, and 20 μm on the bottom. **h** H&E, Masson, and Oil Red O staining of atherosclerotic plaques in aortic roots of *Apoe*^−/−^ mice untreated or infected with *Pg* and/or administrated with vehicle or liposomal clodronate (LC) for 8 weeks. Scale bar = 200 μm in H&E, scale bar = 100 μm in the rest images. **i** Quantitative analyses of plaque size, necrotic area, collagen content, Oil Red O^+^ area, thickness of fibrous cap of the plaques. *n* = 6 per group. **j** The ratio of PI^+^ cells in macrophages over a time course of stimulation with or without ox-LDL (60 μg/mL) and/or *Pg* (MOI = 100). *n* = 4 per group. **k** Flow cytometry analyses of the ratio of necrotic (PI^+^Annexin V^+^) and apoptotic (PI^−^Annexin V^+^) macrophages under ox-LDL (60 μg/mL) and/or *Pg* (MOI = 100) treatment. *n* = 4 per group. **l** The ratio of PI^+^ cells in ox-LDL loaded macrophages pre-administrated with Z-VAD-FMK (5 μM), Nec-1 (10 μM), ferrostatin-1 (FER-1, 5 μM), or VX765 (50 μM) and infected with or without *Pg* (MOI = 100). *n* = 4 per group. The co-localization of p-MLKL and CD68 or F4/80-labled macrophages in the atherosclerotic plaques of human coronary arteries (**m**), rabbit aortic arches (**n**), and mouse aortic roots (**o**). Nuclear DNA (blue) was counterstained with DAPI. Scale bar = 100 μm. Results were represented as mean ± SEM (**c**, **e**, **g**, **i**) or mean ± SD (**j**, **k**, **l**). Data were analyzed by non-parametric Spearman’s test (**a**), one-way ANOVA (**c**, **i**, **k**), unpaired Student t test (**e**) and two-way ANOVA (**g**, **j**, **l**). ****P < 0.0001; ***P < 0.001; **P < 0.01; *P < 0.05

From the analyses of plaque vulnerability, we noticed that the plaque necrotic area exhibited the most significant variation among the examined features (Fig. 1), and even showed a positive association (*r* = 0.7902, *P* < 0.0001) with the number of *Pg* fluorescent clusters within human atherosclerotic plaques, as determined by the non-parametric Spearman’s test (Fig. 2a). Therefore, we further explored *Pg*’s pathogenic role in plaque necrosis. Necrotic core formation is mainly attributed to the demise of VSMCs and foam cells, which primarily derive from macrophages and VSMCs.^36,37^ In human atherosclerotic plaques, we observed that *Pg* was predominantly localized around the CD45^+^CD68^+^ macrophages (Supplementary Fig. 6a). Consistent with the observed increase in the necrotic area (Fig. 1 and Supplementary Figs. 2, 3), a larger proportion of terminal deoxynucleotidyl transferase dUTP nick end labeling (TUNEL)-positive macrophages were detected within aortic atherosclerotic plaques in human samples from high group (Fig. 2b, c), as well as in *Pg*-inoculated rabbits and *Apoe*^−/−^ mice (Fig. 2d–g). Additionally, TUNEL-positive VSMCs was increased in human samples from high group but remained unaltered in rabbits and *Apoe*^−/−^ mice (Supplementary Fig. 6b–d). To further delineate the function of macrophages in *Pg*-induced plaque necrosis, we depleted macrophages in *Apoe*^−/−^ mice via intraperitoneal administration of liposomal clodronate (LC) (Supplementary Fig. 6e). Compared to the vehicle-treated mice, those injected with LC indeed exhibited resistance to *Pg*-induced plaque destabilization, manifested by reduced lesion burden and necrotic core in the aortic roots (Fig. 2h, i). These results emphasize that macrophages are necessary for *Pg*-promoted plaque necrosis and the subsequent development of plaque instability.

Next, we undertook in vivo investigations to validate the impact of *Pg* stimulation on macrophage death. To mimic foam cells, macrophages were preloaded with ox-LDL (60 μg/mL). Ox-LDL-loaded macrophages exhibited a higher rate of cell death compared to PBS-treated control cells, and this effect was further reinforced by *Pg* (MOI = 100) stimulation in a time-dependent manner (Fig. 2j). Notably, *Pg* challenge evoked a pronounced elevation in necrotic cells (Annexin V^+^PI^+^) alongside a concomitant decline in apoptotic cell (Annexin V^+^PI^−^) proportions (Fig. 2k). These findings indicate that *Pg* promotes necrosis, rather than apoptosis, in lipid-laden macrophages. Given that macrophages can experience various regulated necrosis, like ferroptosis, necroptosis, and pyroptosis during atherosclerosis progress, we employed distinct inhibitors to determine which pathway was predominantly affected. Flow cytometry analyses revealed that necrostatin-1 (Nec-1, 10 μΜ) efficiently suppressed *Pg*-induced death of lipid-laden macrophages (Fig. 2l), suggesting necroptosis the primary form of necrosis triggered by *Pg* infection. Necroptosis execution relies on mixed lineage kinase domain-like pseudokinase (MLKL) phosphorylation. We subsequently assessed and observed a significant upregulation of phosphorylated-MLKL (p-MLKL) expression in lipid-laden macrophages exposed to *Pg*, as well as in plaque macrophages from *Pg-*enriched human coronary arteries, *Pg*-infected rabbits, and *Apoe*^−/−^ mice (Fig. 2m–o and Supplementary Fig. 6f–h). Collectively, these findings indicate that *Pg* exposure induces necroptosis in lipid-laden macrophages, potentially predisposing the expansion of the necrotic core in atherosclerosis.

### Oxidative stress serves as a principal driver of *Pg*–induced necroptosis

**Fig. 3:**
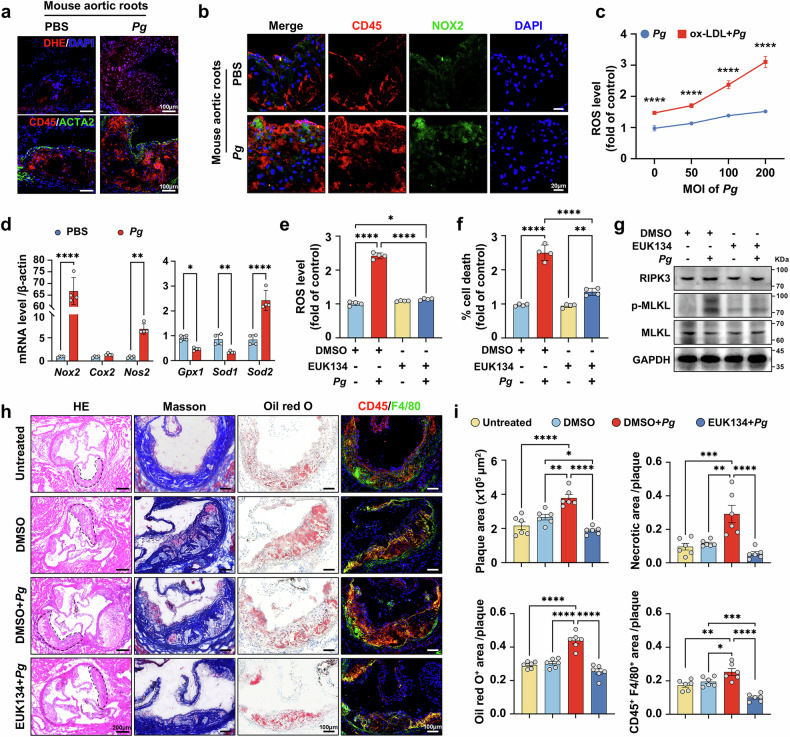
Oxidative stress serves as a principal driver of *Pg*–induced necroptosis. **a** Dihydroethidium (DHE) staining and CD45-ACTA2 co-staining of serial sections of the aortic roots isolated from *Apoe*^–/–^ mice infected with or without *Pg* for 8 weeks. Nuclei were labeled using DAPI. Scale bar = 100 μm. **b** The co-localization of NOX2 and CD45-labled macrophages in the aortic root plaques of *Apoe*^–/–^ mice infected with or without *Pg* for 8 weeks. Nuclei were labeled using DAPI. Scale bar = 20 μm. **c** Flow cytometry analyses of the reactive oxygen species (ROS) level in macrophages infected by *Pg* in different MOIs, and treated with or without ox-LDL (60 μg/mL). *n* = 4 per group. **d** Relative mRNA expression levels of *Nox2*, *Cox2*, *Nos2*, *Gpx1*, *Sod1*, and *Sod2* in ox-LDL (60 μg/mL)-loaded macrophages treated with or without *Pg* (MOI = 100) for 24 h. *n* = 4 per group. Flow cytometry analyses of ROS production (**e**) and ratio of PI^+^ cells (**f**) in ox-LDL (60 μg/mL)-loaded macrophages pre-administrated with EUK134 (10 μM) and infected with *Pg* (MOI = 100). *n* = 4 per group. **g** Western blot analyses of RIPK3, p-MLKL, MLKL, and GAPDH expression in ox-LDL (60 μg/mL)-loaded macrophages pre-administrated with EUK134 (10 μM) and infected with *Pg* (MOI = 100) for 24 h. GAPDH was used as the loading control. **h** H&E, Masson, Oil Red O staining, and CD45 and F4/80 co-immunohistochemical staining of atherosclerotic plaques in aortic roots of *Apoe*^−/−^ mice untreated or challenged by *Pg* under the administration of EUK134 for 8 weeks. Scale bar = 200 μm in H&E, scale bar = 100 μm in the rest images. **i** Quantitative analyses of plaque size, necrotic area, Oil Red O^+^ area, and CD45 and F4/80-positive areas of the plaques in **h**. *n* = 6 per group. Results were represented as mean ± SD (**c**, **d**, **e**, **f**) or mean ± SEM (**i**). Data were analyzed by two-way ANOVA (**c**, **d**), or one-way ANOVA (**e**, **f**, **i**). ****P < 0.0001; ***P < 0.001; **P < 0.01; *P < 0.05

Oxidative stress is a well-documented mediator of macrophage necroptosis.^38^ To investigate its contribution to *Pg*-enhanced macrophage necroptosis, we examined the degree of cellular oxidative stress upon *Pg* infection both in vivo and in vitro. In *Pg*-infected *Apoe*^−/−^ mice, macrophage-derived reactive oxygen species (ROS) and the expression of NADPH oxidase 2 (NOX2), an enzyme involving in ROS generation, were significantly increased within the aortic root plaques (Fig. 3a, b). Consistently, in *Pg* co-cultured macrophages, ROS production increased proportionally with escalating multiplicities of infection (MOIs) and was further exacerbated when preloaded with ox-LDL (Fig. 3c). The genes involved in ROS production, such as *NOX2*, nitric oxide synthase 2 (*Nos2*), were also upregulated in mRNA expression, while genes associated with antioxidant defense, such as superoxide dismutase 1 (*Sod1*) and glutathione peroxidase 1 (*Gpx1*) were downregulated (Fig. 3d). Since ox-LDL is also a potent ROS activator, we then stimulated macrophages with varying concentrations of ox-LDL for 2 h, and observed a corresponding rise in ROS production, with which peaking at 100 μg/mL (Supplementary Fig. 7a). Prolonged ox-LDL stimulation for 24 h resulted in increased macrophage necrosis (Supplementary Fig. 7b), coupled with augmented protein levels of necroptosis executor p-MLKL and receptor-interacting protein kinase-3 (RIPK3) (Supplementary Fig. 7c). However, it is notable that the magnitude of this effect was lower than that induced by concurrent *Pg* and ox-LDL exposure. In addition, macrophages were stimulated with native LDL, which was considered less pro-atherosclerotic than ox-LDL.^39^ The results exhibited that *Pg* slightly enhanced native LDL uptake and ROS level, and had a negligible impact on LDL-induced cell death (Supplementary Fig. 7d–f). To further validate the impact of oxidative stress, ox-LDL-loaded macrophages were pretreated with the antioxidant EUK-134 (10 μM), which effectively diminished *Pg*-evoked ROS generation comparable to control levels (Fig. 3e). This ROS reduction correspondingly decreased *Pg*-induced macrophage necrosis (Fig. 3f), as well as p-MLKL and RIPK3 protein levels (Fig. 3g). In general, our findings suggest that oxidative stress is an essential molecular driver of *Pg*-induced necroptosis in lipid-laden macrophages. Moreover, it is noteworthy that ox-LDL-induced ROS production and cell death were further intensified by *Pg* inoculation, suggesting a potential crosstalk between ox-LDL and *Pg*.

To better clarify how oxidative stress impacts *Pg*-promoted plaque instability, *Apoe*^−/−^ mice were intervened with EUK134. Compared to *Pg*-inoculated mice, mice treated with EUK134 exhibited a significant alleviation in the plaque instability, as evidenced by a decrease in necrotic core, Oil red O-positively stained area, and the proportion of CD45^+^F4/80^+^ macrophages within the plaques (Fig. 3h, i). Our results further confirm that oxidative stress exacerbates plaque instability in *Pg*-infected animals.

### The *Pg*-amplified ROS production and necroptosis do not solely depend on LPS

Next, we aimed to identify the key components of *Pg* responsible for trigging macrophage oxidative stress. Considering the well-characterized pro-oxidative properties of LPS in macrophages,^40^ we first examined the effects of *Pg*-LPS. Treatment with 1 μg/mL *Pg*-LPS led to significant ROS production and augmented cell necrosis in macrophages (Supplementary Fig. 8a, b); however, both remained lower than in *Pg*-infected macrophages. To further confirm the role of the LPS in *Pg*-induced oxidative stress, we inhibited the TLR4 signaling pathway using resatorvid (TAK242, 2 μM). Macrophages treated with TAK242 under *Pg* exposure manifested a considerable reduction of ROS production and cell death, though both levels remained elevated versus controls (Supplementary Fig. 8c, d). A similar pattern was observed in p-MLKL protein levels (Supplementary Fig. 8e). Our findings imply that besides LPS, other *Pg*-derived components appear to drive macrophage oxidative stress and subsequent necroptosis.

### Gingipains play important roles in *Pg*-aggravated oxidative stress

**Fig. 4:**
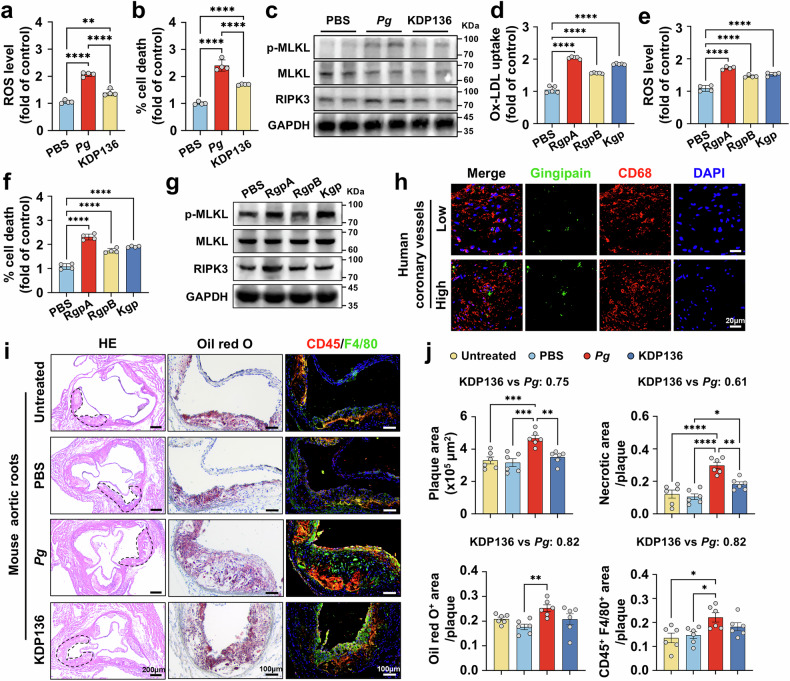
Gingipains play important roles in *Pg*-aggravated oxidative stress. Flow cytometry analyses of the ROS production (**a**) and the ratio of PI^+^ cells (**b**) in ox-LDL (60 μg/mL) loaded macrophages infected with *Pg* or KDP136 (MOI = 100). *n* = 4 per group. **c** Western blot analyses of RIPK3, p-MLKL, MLKL, and GAPDH expression in ox-LDL (60 μg/mL)-loaded macrophages infected with *Pg* or KDP136 (MOI = 100) for 24 h. GAPDH was used as the loading control. Flow cytometry analyses of the ox-LDL uptake (**d**), the ROS production (**e**), and the ratio of PI^+^ cells (**f**) in ox-LDL-loaded macrophages exposed to RgpA, RgpB, or Kgp (1 μg/mL). *n* = 5 per group in (**d**), *n* = 4 per group in (**e**, **f**). **g** Western blot analyses of RIPK3, p-MLKL, MLKL, and GAPDH expression in ox-LDL (60 μg/mL)-loaded macrophages treated with RgpA, RgpB, or Kgp (1 μg/mL) for 24 h. GAPDH was used as the loading control. **h** The co-staining of RgpA and CD68 (macrophage marker) in the coronary plaques of human. Scale bar = 20 μm. **i** H&E, Oil Red O staining, and CD45 and F4/80 co-staining of the aortic root plaques from *Apoe*^−/−^ mice infected with or without *Pg* or KDP136 for 8 weeks. Scale bar = 200 μm in H&E, scale bar = 100 μm in the rest images. **j** Quantitative analyses of plaque size, necrotic area, Oil Red O^+^ area, CD45, and F4/80-positive areas of the plaques in **i**. *n* = 6 per group. Results were presented as mean ± SD (**a**, **b**, **d**, **e**, **f**) or mean ± SEM (**j**). All data were analyzed by one-way ANOVA. ****P < 0.0001; ***P < 0.001; **P < 0.01; *P < 0.05

Gingipains, comprising arginine-gingipain A (RgpA), arginine-gingipain B (RgpB), and lysine-gingipain (Kgp), are cysteine proteases secreted by *Pg* and constitute *Pg*’s principal pathogenic determinants. To elucidate the contribution of gingipains to macrophage oxidative stress and necroptosis, we utilized the gingipains (Rgps and Kgp)–deficient *Pg* mutant strain KDP136. In ox-LDL-preloaded macrophages, *Pg* inoculation led to a pronounced escalation of ROS generation and cell death compared to unstimulated cells, whereas KDP136 induced a more moderate response in both aspects (Fig. 4a, b). Additionally, *Pg* significantly enhanced MLKL phosphorylation and RIPK3 expression in macrophages, while KDP136 resulted in a slight elevation (Fig. 4c), suggesting that gingipains are vital mediator in *Pg*-induced oxidative stress and necroptosis. To further validate this, recombinant gingipains were applied. With the existence of ox-LDL, gingipains (1 μg/mL) markedly promoted ox-LDL accumulation, ROS production, and necroptosis of macrophages (Fig. 4d–g). Moreover, gingipains were detected both within and around the macrophages in atherosclerotic plaques from human coronary arteries and *Pg*-inoculated rabbits and *Apoe*^−/−^ mice (Fig. 4h and Supplementary Fig. 9a, b). Consistent with the above findings, *Apoe*^−/−^ mice infected with KDP136 exhibited alleviated plaque instability compared to those infected with *Pg*, as evidenced by smaller necrotic core and plaque size, and lowered macrophage p-MLKL protein levels (Fig. 4i, j and Supplementary Fig. 9c). Collectively, these results demonstrate gingipains as important contributors to *Pg*-aggravated macrophage oxidative stress within atherosclerotic plaques.

### *Pg*-enlarged oxidative stress mostly results from enhanced MSR1-mediated ox-LDL uptake

**Fig. 5:**
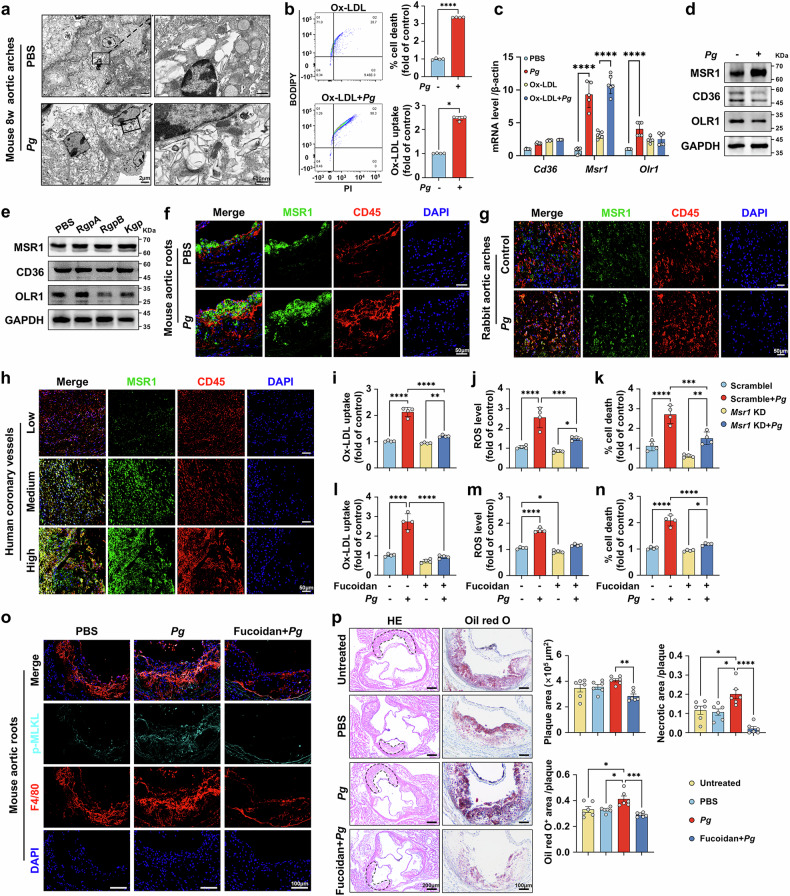
*Pg*-enlarged oxidative stress mostly results from enhanced MSR1-mediated ox-LDL uptake. **a** TEM images of the aortic arch plaque of *Apoe*^−/−^ mice infected with or without *Pg* for 8 weeks. Scale bar = 2 μm on the left, and 500 nm on the right. **b** Flow cytometry analyses of the ratio of PI^+^ macrophages and the ox-LDL accumulation in PI^+^ macrophages under ox-LDL (60 μg/mL) and *Pg* (MOI = 100) challenge for 24 h. *n* = 4 per group. **c** qRT-PCR analyses of *Cd36*, *Msr1*, *Olr1* expression in macrophages treated with or without ox-LDL (60 μg/mL) and/or *Pg* (MOI = 100) for 24 h. β-actin was used as control. *n* = 4–6 per group. Western blot analyses of MSR1, CD36, OLR1, or GAPDH expression in ox-LDL (60 μg/mL)-loaded macrophages treated with *Pg* (MOI = 100) (**d**), or RgpA, RgpB, or Kgp (1 μg/mL) (**e**) for 24 h. GAPDH was used as the loading control. The co-localization of MSR1 and CD45-labled macrophages in the plaques of *Apoe*^–/–^ mice aortic roots (**f**), rabbit aortic arches (**g**), and human coronary vessels (**h**). Scale bar = 50 μm. Flow cytometry analyses of the ox-LDL uptake (**i**), ROS production (**j**), and the ratio of PI^+^ cells (**k**) in *Msr1*-knockdown macrophages loaded with ox-LDL (60 μg/mL) and infected with *Pg* (MOI = 100). *n* = 4 per group. Flow cytometry analyses of the ox-LDL uptake (**l**), ROS production (**m**), and the ratio of PI^+^ cells (**n**) in fucoidan (40 μg/mL) pretreated macrophages loaded with ox-LDL (60 μg/mL) and challenged by *Pg* (MOI = 100). *n* = 4 per group. **o** The co-staining of p-MLKL and F4/80 (macrophage marker) in the aortic root plaques from *Apoe*^−/−^ mice treated with fucoidan and/or *Pg* for 8 weeks. Scale bar = 100 μm. **p** H&E and Oil Red O staining of aortic root plaques from *Apoe*^−/−^ mice treated with fucoidan and/or *Pg* for 8 weeks. Quantitation of the plaque size, as well as the ratios of necrotic area and Oil Red O^+^ area to plaque area were at right. *n* = 6 per group. Scale bar = 200 μm in H&E, scale bar = 100 μm in Oil Red O. Results were expressed as mean ± SD (**b**, **c**, **i**–**n**) or mean ± SEM (**p**). Data were analyzed by unpaired two-tailed student *t*-test (**b**), two-way ANOVA (**c**) or one-way ANOVA (**i**–**n**, **p**). ****P < 0.0001; ***P < 0.001; **P < 0.01; *P < 0.05

Considering that the ROS generation and cell necroptosis evoked by ox-LDL could be significantly exacerbated under *Pg* inoculation, we hypothesized a potential interplay between *Pg* and ox-LDL. Notably, we observed numerous dying macrophages laden with abundant lipid droplets surrounding the necrotic cores within the aortic arch plaques from *Pg*-infected *Apoe*^−/−^ mice (Fig. 5a). In vitro experiments revealed that not only did the proportion of necrotic cells rise, but the ox-LDL deposition in necrotic cells was also significantly higher in *Pg*-infected macrophages than the non-*Pg*-infected cells (Fig. 5b), implying a potential involvement of ox-LDL accumulation in *Pg*-induced cell necrosis. To further investigate this, macrophages were treated with *Pg* in distinct MOIs for 24 h under ox-LDL exposure. *Pg* markedly increased intracellular lipid deposition at MOIs from 10 to 1000, reaching its maximum effect at an MOI of 100 (Supplementary Fig. 10a). Similarly, Oil red O staining confirmed that *Pg* aggravated the lipid burden in macrophages (Supplementary Fig. 10b). Since lipid deposition regulated by two key processes—lipid uptake and efflux—we focused on elucidating which process *Pg* affects in macrophage lipid accumulation. When co-cultured with ox-LDL, macrophages exposed to *Pg* exhibited a notable increase in intracellular cholesterol concentrations (Supplementary Fig. 10c) and a slight decrease in cholesterol efflux (Supplementary Fig. 10d) compared to the control group. This indicate that *Pg* primarily influences the lipid uptake process rather than the lipid efflux in macrophages. To better understand how augmented lipid uptake correlates with *Pg*-induced ROS production and cell necrosis, we applied (2-hydroxypropyl)-β-cyclodextrin (HP-β-CD) to unload the intracellular cholesterol in macrophages. The data revealed that treatment with 10 mM HP-β-CD effectively suppressed the intracellular cholesterol content, ROS levels, and necroptosis of macrophages infected by *Pg* (Supplementary Fig. 10e–h). However, administration of necroptosis inhibitor Nec-1 (10 μM) failed to suppress the lipid uptake (Supplementary Fig. 10i). Prior research revealed that ox-LDL can enhance RIPK3 and MLKL expression, leading to necroptosis, which can be prevented by scavenging cellular ROS.^41^ Consistent with these findings, our results suggest that *Pg* significantly facilitates macrophage lipid uptake, which may serve as the underlying mechanism aggravating *Pg*-induced macrophage oxidative stress and the further necroptosis.

To identify the specific internalization pathways through which *Pg* influences macrophage ox-LDL uptake, we examined three well-characterized mechanisms: the receptor-mediated (clathrin-dependent) pathway, lipid raft-/caveolin-dependent pathway, and macropinocytosis. Macrophages were treated with pathway-specific inhibitors, including dynasore (10 μM), filipin (5 μM), and LY294002 (50 μM), to observe their effects on ox-LDL uptake. Inhibition with dynasore almost completely abolished lipid uptake by macrophages, regardless of *Pg* treatment. In contrast, Filipin and LY294002 only partially lessened macrophage lipid uptake, and failed to counteract the enhancing effect of *Pg* (Supplementary Fig. 10j). Furthermore, dynasore also significantly diminished the ROS production and cell death of macrophages (Supplementary Fig. 10k, l). These results demonstrate that *Pg* mainly enhances macrophage lipid uptake through the receptor-mediated internalization pathway.

Thereafter, the expression levels of critical receptors participating in receptor-mediated internalization, including MSR1, oxidized low-density lipoprotein receptor 1 (OLR1) and scavenger receptor class B (CD36) were assessed, and MSR1 and OLR1 were significantly upregulated in *Pg*-cultured macrophages (Fig. 5c). Notably, upon ox-LDL exposure, the upregulation of MSR1 became more pronounced, whereas OLR1 expression remained unchanged (Fig. 5c, d). Gingipains also markedly elevated MSR1 expression in ox-LDL loaded macrophages (Fig. 5e). Consistently, the increased MSR1 expression was observed in macrophages within atherosclerotic plaques of *Pg*-treated mice and rabbits, as well as in the human coronary plaques from high group (Fig. 5f–h). To further elucidate the role of MSR1, we knocked down (KD) its expression in macrophages, followed by ox-LDL stimulation and *Pg* inoculation. MSR1 KD resulted in remarkably reduced lipid engulfment, ROS production, and following necroptosis in *Pg*-infected macrophages (Fig. 5i–k and Supplementary Fig. 10m). Similarly, treatment with MSR1 antagonist fucoidan (40 μg/mL) effectively mitigated lipid accumulation, ROS generation, and cell death in *Pg* co-cultured macrophages (Fig. 5l–n). Systemic delivery of fucoidan in *Pg*-inoculated *Apoe*^−/−^ mice substantially suppressed macrophages necroptosis, and virtually ablated the effects of *Pg* on necrotic core and plaque instability (Fig. 5o, p). In conclusion, our results suggest that *Pg* and its gingipains enhance *Msr1* transcription and MSR1-mediated lipid uptake, driving excessive ROS formation and ultimately contributing to macrophage death.

### Proteolysis of FOXO3 by gingipains upregulates the transcription of MSR1

**Fig. 6:**
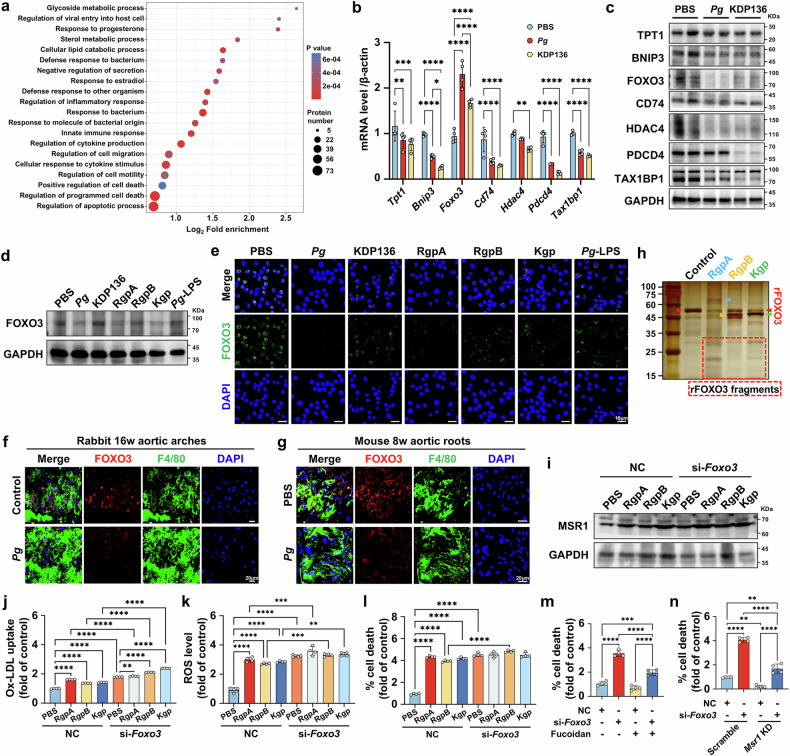
Proteolysis of FOXO3 by gingipains upregulates the transcription of MSR1. **a** GO enrichment analysis of differential proteins between ox-LDL (60 μg/mL)-loaded macrophages treated with *Pg* (MOI = 100) versus PBS for 24 h (*n* = 4 per genotype). *P* < 0.05 represented statistically significant difference, and upregulated or downregulated proteins were identified with a fold-change (*Pg*/PBS) > 1.15 or <0.87. qRT-PCR (**b**) and Western blot (**c**) analyses of TPT1, BNIP3, FOXO3, CD74, HDAC4, PDCD4, and TAX1BP1 expression in ox-LDL (60 μg/mL)-loaded macrophages infected with *Pg* or KDP136 (MOI = 100) for 24 h. β-actin was used as control, and *n* = 4 per group (**b**). GAPDH was employed as the loading control in (**c**). Western blot analyses (**d**) and immunohistochemical staining (**e**) of FOXO3 in ox-LDL (60 μg/mL)-loaded macrophages infected with *Pg*, KDP136 (MOI = 100), RgpA, RgpB, Kgp, or *Pg*-LPS (1 μg/mL) for 24 h. GAPDH was employed as the loading control in (**d**). Scale bar = 10 μm in (**e**). The co-staining of FOXO3 and F4/80 (macrophage marker) in the plaques of rabbit aortic arches (**f**), and mouse aortic roots (**g**). Scale bar = 20 μm. **h** Silver staining of recombinant human FOXO3 (rFOXO3, 207.9 μM) incubated with RgpA, RgpB or Kgp (2.1 μM) for 60 min at 37 °C. Red arrowhead points to original rFOXO3. Blue arrowhead points to RgpA. Orange arrowhead points to RgpB. Green arrowhead points to Kgp. Dashed lines encircle rFOXO3 fragments. **i** Western blot analyses of MSR1 expression in *Foxo3* siRNA (si-*Foxo3*) transfected macrophages loaded with ox-LDL (60 μg/mL) and stimulated with RgpA, RgpB, or Kgp (1 μg/mL) for 24 h. GAPDH was used as control. Flow cytometry analyses of the ox-LDL uptake (**j**), ROS production (**k**), and the ratio of PI^+^ cells (**l**) in si-*Foxo3* transfected macrophages treated with RgpA, RgpB, or Kgp (1 μg/mL) in the presence of ox-LDL (60 μg/mL). *n* = 4 per group. **m** Flow cytometry analyses of the ratio of PI^+^ cells in si-*Foxo3* transfected macrophages pre-administrated with or without fucoidan (40 μg/mL) and exposed to ox-LDL (60 μg/mL) for 24 h. *n* = 4 per group. **n** Flow cytometry analyses of the proportion of PI^+^ cells in *Msr1* and/or *Foxo3* knockdown macrophages treated with ox-LDL (60 μg/mL) for 24 h. *n* = 4 per group. Results were expressed as mean ± SD. Data were analyzed by two-way ANOVA (**b**) or one-way ANOVA (**j–n**). ****P < 0.0001; ***P < 0.001; **P < 0.01; *P < 0.05

To explore the underlying mechanism by which gingipains upregulate *Msr1* transcription, we conducted TMT-labeled quantitative proteomics to profile protein expression in *Pg*-exposed macrophages compared to control cells. 93 differentially expressed proteins were identified, and the majority of them were enriched in cell death-related pathways (Fig. 6a). Screening the genes involving in the necrosis-related pathways, we analyzed the mRNA and protein levels of those related to transcriptional regulation. Our results revealed that translationally-controlled tumor protein (*Tpt1*), BCL2 interacting protein 3 (*Bnip3*), cluster of differentiation 74 (*Cd74*), histone deacetylase 4 (*Hdac4*), programmed cell death protein 4 (*Pdcd4*) and Tax1-binding protein 1 (*Tax1bp1*) presented decreased mRNA and protein levels in both *Pg-* and KDP136-infected macrophages (Fig. 6b, c). However, FOXO3 presented upregulated mRNA expression but reduced protein level under *Pg* infection, which recovered under KDP136 inoculation (Fig. 6b, c). Given that gingipains, as proteases derived from *Pg*, exhibit broad-spectrum protein cleavage activity,^42^ we postulated FOXO3 as a potential direct substrate. This hypothesis was supported by dose-dependent reduction in FOXO3 protein levels in *Pg*-infected macrophages (Supplementary Fig. 11a). Similarly, a comparable decline in FOXO3 levels occurred in gingipains-exposed macrophages but was absent in both PBS- and KDP136-treated macrophages (Fig. 6d, e). In *Pg*-inoculated rabbits and *Apoe*^−/−^ mice, FOXO3 protein levels in plaque macrophages were also decreased (Fig. 6f, g). To functionally validate FOXO3 as a gingipain substrate, silver staining was employed and confirmed the proteolytic degradation of recombinant FOXO3 by RgpA, RgpB, and Kgp, yielding cleavage fragments of varying sizes (Fig. 6h). Additionally, in *Pg*-LPS-infected macrophages, a slight decrease in FOXO3 protein level was detected (Fig. 6d, e and Supplementary Fig. 11b). Prior studies evidenced that FOXO3 can be phosphorylated through extracellular signal-regulated kinase (ERK) pathway, with ensuing nuclear export and ultimate cytoplasmic degradation.^43^ In our study, we observed *Pg*-LPS-activated mitogen-activated protein kinase (MAPK)/ERK signal channeling, along with enhanced FOXO3 phosphorylation, as well as concomitant reduction in FOXO3 protein abundance (Supplementary Fig. 11b), implicating that *Pg*-LPS may also contribute to FOXO3 degradation.

Furthermore, we silenced FOXO3 with siRNA in macrophages and observed increased MSR1 expression, enhanced ox-LDL uptake, elevated ROS production, and greater cell death, with gingipain stimulation causing only a slight additional increase. (Fig. 6i–l). Notably, fucoidan efficiently attenuated macrophage death in *Foxo3*-KD cells (Fig. 6m). Meanwhile, in *Msr1*-KD macrophages, *Foxo3*-KD resulted in a less pronounced increase in cell death (Fig. 6n). Taken together, these findings demonstrate that *Pg* and gingipains reduce macrophage FOXO3 protein level to upregulate MSR1 transcription.

### HDAC2 is recruited to the promoter by FOXO3 and inhibits MSR1 transcription

**Fig. 7:**
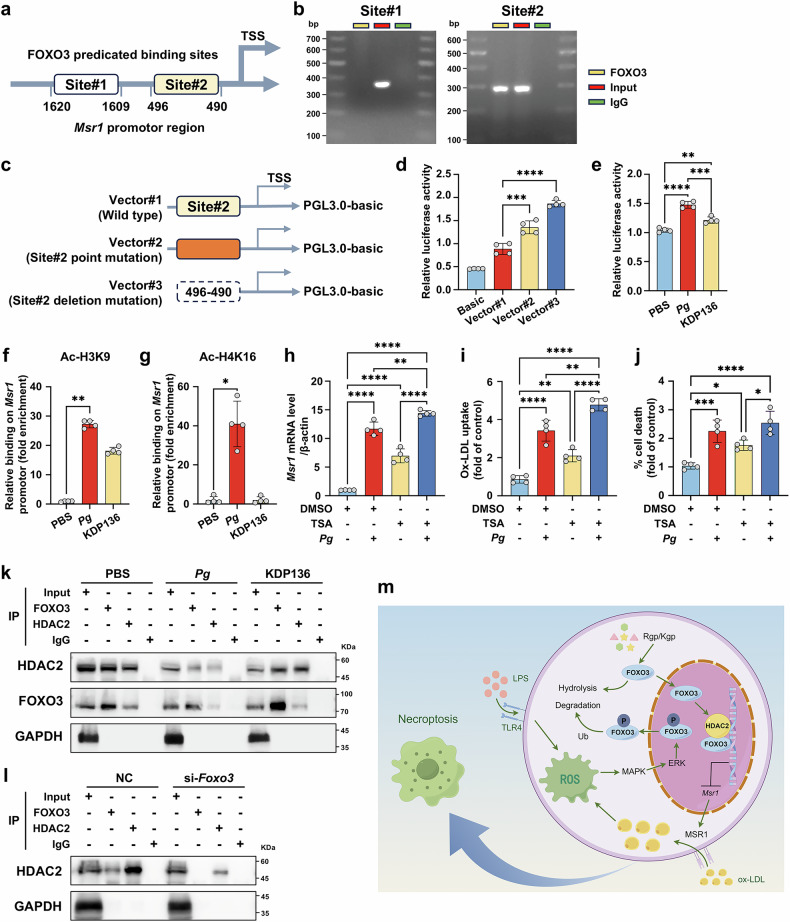
HDAC2 is recruited to the promoter by FOXO3 and inhibits MSR1 transcription. **a** Potential FOXO3 binding sites on mouse *Msr1* promoter predicted by JASPAR database. **b** CHIP was conducted using FOXO3 antibody and IgG (negative control). **c** Vector information of the wild-type *Msr1* promoter (−496 /−490) and its mutation/depletion mutants. **d** Dual luciferase reporter assay of the activities of the wild-type *Msr1* promoter, and its mutation and depletion mutants in macrophages loaded with ox-LDL (60 μg/mL). *n* = 4 per group. **e** Dual luciferase reporter assay of the activities of the wild-type *Msr1* promoter in macrophages loaded with ox-LDL (60 μg/mL) and infected with *Pg* or KDP136 (MOI = 100). *n* = 4 per group. **f**, **g** CHIP-PCR analyses of the histone H3 and histone H4 acetylation level in ox-LDL (60 μg/mL)-loaded macrophages treated with *Pg* or KDP136 (MOI = 100). *n* = 4 per group. qRT-PCR analyses of *Msr1* expression (**h**), flow cytometry analyses of the ox-LDL uptake (**i**) and the ratio of PI^+^ cells (**j**) in ox-LDL (60 μg/mL)-loaded macrophages pre-administrated with TSA (1 μM) and challenged with *Pg* (MOI = 100) for 24 h. *n* = 4 per group. **k** FOXO3 and HDAC2 binding detection by Co-IP in ox-LDL (60 μg/mL) loaded macrophages infected with *Pg* or KDP136 (MOI = 100) for 24 h. **l** FOXO3 and HDAC2 binding detection by Co-IP in si-*Foxo3* transfected macrophages upon ox-LDL (60 μg/mL) stimulation for 24 h. **m** Schematic diagram showing the mechanism of *Pg*-promoted macrophage necroptosis. In macrophages, gingipains secreted by *Pg* directly hydrolyze FOXO3 in the cytoplasm, thereby inhibiting its nuclear translocation. Additionally, *Pg*-LPS activates MAPK/ERK-mediated phosphorylation of FOXO3, promoting its nuclear export, and subsequent ubiquitination (Ub) and degradation. The reduction in FOXO3 levels diminishes HDAC2 binding to the MSR1 promoter and increases promoter acetylation, leading to upregulation of MSR1 expression. This enhanced MSR1 expression promotes lipid uptake and the following oxidative stress, ultimately triggering macrophage necroptosis. Diagram is generated by figdraw.com. Results were represented as mean ± SD. Data were analyzed by one-way ANOVA (**d**, **e**, **h**, **i**, **j**), or Kruskal–Wallis test (**f**, **g**). ****P < 0.0001; ***P < 0.001; **P < 0.01; *P < 0.05

Next, we investigated how FOXO3 inhibits *Msr1* transcription. According to the JASPAR database, there were 2 predicted potential binding sites of FOXO3 in mouse *Msr1* promoter (Fig. 7a). Chromatin immunoprecipitation (ChIP) and dual-luciferase assays verified that FOXO3 exhibited specific binding affinity for the *Msr1* promoter region at site#2 (−496/−490), repressing its transcription (Fig. 7b–d). *Pg* inoculation enhanced *Msr1* transcription of lipid-laden macrophages, whereas KDP136 showed an attenuated effect (Fig. 7e). In human *MSR1* promoter, a predicted FOXO3 binding site was identified (Supplementary Fig. 12a). ChIP assay further validated that FOXO3 selectively binds to the *MSR1* promoter (−905/−898) in human macrophages (Supplementary Fig. 12b). Given the HDAC2 can selectively regulate FOXO3-mediated gene transcription during oxidative stress,^44^ we proposed that *Pg*-induced *Msr1* transcription upregulation might result from enhanced *Msr1* promoter acetylation. As expected, histone acetylation levels at the *Msr1* promoter locus were significantly elevated in *Pg*-challenged macrophages versus controls (Fig. 7f, g). Treatment with trichostatin A (TSA, 1 μM), a specific HDAC inhibitor, contributed to amplified *Msr1* transcription, along with the augmented lipid uptake and cell death (Fig. 7h–j). The co-immunoprecipitation (Co-IP) assay further verified the interaction between FOXO3 and HDAC2 in wild type (WT) macrophages, while this association was weakened upon *Pg* inoculation, but restored under KDP136 infection (Fig. 7k). Similarly, when FOXO3 was KD, it failed to associate with HDAC2 (Fig. 7l). Beyond *Msr1*, FOXO3 also bound to the promoters of other genes related to necroptosis, like caspase-6 (*Casp6*), catalase (*Cat*) and tumor necrosis factor ligand superfamily member 10 (*Tnfsf10*). However, this interaction was also diminished in *Pg*-stimulated macrophages (Supplementary Fig. 12c). Collectively, our evidences reveal that HDAC2 is recruited to the *Msr1* promoter by FOXO3, thereby repressing *Msr1* transcription (Fig. 7m).

## Discussion

Atherosclerosis is central to the pathology of ASCVDs, a group of diseases characterized by arterial occlusion due to atheroma formation. The rupture of atheroma can trigger various pathological cardiovascular events, including MI and ischemic stroke, which is also termed acute coronary syndrome (ACS). Epidemiological, histopathological, and interventional evidences have established periodontal diseases as a non-traditional but independent contributor to both ACS and atherosclerosis.^11,45^ A large body of animal model research has conclusively proven that periodontal pathogens significantly accelerate atherosclerosis development and progression.^46,47^ Our previous study also demonstrated that *Pg* aggravates atherosclerosis.^15^ However, the impact of periodontal pathogens on the vulnerability of atherosclerotic lesions has largely been overlooked. Although existing studies point to a link between *Pg* and plaque instability,^19,48^ the underlying biological mechanisms remain poorly understood.

Vulnerable plaques, clinically synonymous with “high-risk plaques”, refer to atherosclerotic lesions with structural instability predisposing to rupture and thrombosis, ultimately causing acute cardiovascular events or death. Their key histopathological features encompass a large necrotic core covered by a thin fibrous cap, a dense macrophage distribution, expansive remodeling that preserves the lumen, pathological neovascularization, IPH, adventitial inflammatory infiltration, and spotty calcification.^32^ Earlier investigations exploring *Pg*-related plaque destabilization predominantly focused on VSMCs, macrophages, and collagen content within the plaque,^19^ which may not provide a comprehensive assessment. In our study, a broader range of features were measured, including necrotic core area, lipid deposition, macrophage infiltration, collagen remodeling, fibrous cap thickness, and IPH. We found that human atherosclerotic plaques enriched with higher levels of *Pg* exhibited increased plaque vulnerability, characterized by a larger necrotic area, a higher frequency of IPH and more macrophage infiltration. In *Pg*-infected rabbits and *Apoe*^−/−^ mice, an enlarged necrotic core was also observed, while fibrous cap thickness remained unchanged. These results thus confirm that *Pg* contributes to plaque vulnerability, and further support periodontitis as a risk factor for ACS. However, this study has certain limitations. The *Apoe*^−/−^ mice are widely used animal models for atherosclerosis, and their advanced plaques recapitulate critical histopathological characteristics of human unstable plaques, particularly in terms of a large necrotic core, a thin fibrous cap, neoangiogenesis and the presence of IPH; however, spontaneous plaque rupture occurs rarely in mouse model, leading to infrequent MI and stroke.^3,49^ Despite the proposal of other animal modeling approaches to study atherosclerotic plaque destabilization,^50^ a standardized model remains lacking.

Foam cell and VSMC death accounts substantially for the expansion of necrotic core during atherosclerosis progression. Foam cells primarily originate from macrophages and VSMCs.^36,37^ In this study, we observed increased macrophage death within plaques in *Pg*-enriched human coronary vessels and *Pg*-infected rabbits and *Apoe*^−/−^ mice, while VSMC death remained unchanged. Macrophage death has been established as a critical driver to plaque necrosis,^41^ and can manifest diverse programmed cell death, encompassing apoptosis, ferroptosis, pyroptosis, and necroptosis.^36^ Additionally, it has been reported that *Pg* can cause macrophage apoptosis and pyroptosis.^28^ Here, we found that *Pg* elevated both apoptotic and necrotic macrophage death, and markedly enhanced necroptosis in the presence of ox-LDL, indicating ox-LDL’s synergistic role in *Pg*-evoked macrophage necroptosis. Coincidence with prior research, plaque-retained ox-LDL triggers macrophage necroptosis through RIPK1/RIPK3 activation, resulting in substantial damage-associated molecular patterns liberation that intensifies intraplaque inflammation.^41,51^ Thess processes thus compose a feed-forward loop that exacerbates necrotic core expansion in advanced lesions. The atherosclerotic microenvironment engages diverse cellular participants beyond macrophages and VSMCs, with lymphoid populations, endothelial cells, and neutrophils all contributing to lesion progression through distinct mechanisms.^33–35^ Considering that endothelial dysfunction exerts a more pivotal role in atherogenesis, our present study focuses on advanced plaque destabilization mechanisms, thus excluding analysis of intraplaque endothelial alterations. In our study, fewer VSMCs and more CD3^+^ T cells were observed in human atherosclerotic plaques with higher level of *Pg*, as well as in plaques from *Pg*-infected rabbits, but not in *Pg*-infected mice. The intraplaque neutrophil levels remained unaltered in human, rabbit, and mouse samples. These results implied that VSMCs and T cells may also participate in *Pg*-accelerated atherosclerotic plaque progression, warranting further investigation.

Ox-LDL can initiate multiple downstream signaling cascades,^52^ notably through ROS generation that modulate redox-sensitive transcription factors so as to alter cellular gene expression profiles.^53^ Previous research has established that ox-LDL drives necroptosis via raising the expression of necroptosis-related genes, *MLKL* and *RIPK3*, in a ROS-dependent manner.^41^ Oxidative stress plays a fundamental role in cardiovascular disease, with ROS in macrophages contributing to key pathological processes in atherosclerosis, such as inflammation, lipid uptake, and cell death.^54^ Studies have shown that increased macrophage death via ROS generation resulted in decreased atherosclerotic plaque stability.^55^ In line with these studies, we detected more ox-LDL uptake in *Pg*-infected macrophages due to *MSR1* upregulation, which triggered excessive ROS production and, consequently, necroptosis. Furthermore, we observed that pretreatment with the antioxidant EUK-134 significantly reduced necroptosis in *Pg*-infected macrophages and improved plaque stability. These results provide mechanistic insights into how *Pg* induces more macrophage necroptosis in the presence of ox-LDL.

*Pg*, a Gram-negative anaerobic bacterium, synthesizes cysteine proteases as its dominant virulence determinants, termed gingipains, including Kgp, RgpA, and RgpB. Gingipains are secreted both as soluble enzymes and via outer membrane vesicles (OMVs) into the extracellular environment.^42,56^ Gingipains are fundamentally required for *Pg* survival and implementation of multiple pathogenic processes, including initial host colonization, immune evasion, and tissue destruction.^42^ Previous studies have shown that oral infection with *Pg* significantly promotes atherosclerotic plaque formation compared to infection with KDP136.^57^ Whereas, the effect of gingipains on atherosclerotic plaque instability remains poorly understood. Our research showed that KDP136 infection led to less plaque destabilization than *Pg* infection. Moreover, gingipain stimulation enhanced ox-LDL deposition in macrophages, hence facilitating necroptosis. The protease activity of gingipains not only supports *Pg* for nutritional acquisition but also disrupts host defense mechanisms.^58^ Here, we further identified that gingipains can hydrolyze FOXO3, suggesting a novel mechanism by which *Pg* modulates host cell function.

The FOXO transcription factor family comprises four members in mammalian cells: FOXO1, FOXO3, FOXO4, and FOXO6. They are implicated in diverse homeostatic regulation and pathogenesis underlying cancer, chronic neurological diseases, diabetes, and cardiovascular disorders.^59^ It has been reported that *FoxO* knockout in mice contributed to increased oxidative stress and accelerated atherosclerosis.^60^ Our proteomic analysis revealed that only FOXO3 was differentially expressed in *Pg*-exposed macrophages. FOXO3 protein levels were reduced in *Pg*-inoculated macrophages, as well as within the atherosclerotic plaques of *Pg*-infected rabbits and mice. Further investigation demonstrated that this reduction in FOXO3 protein level is due to direct hydrolysis of gingipains. The activity of FOXO is precisely modulated via various patterns, such as subcellular localization, protein-protein interactions, and post-translational modifications.^61^ Of note, we uncovered an innovative mechanism through which decreased FOXO3 evokes upregulation of MSR1. In WT-macrophages, FOXO3 recruits HDAC2 to the *Msr1* promoter, leading to histone deacetylation and suppression of *Msr1* transcription. *Pg*-induced reduction of FOXO3 diminishes HDAC2 binding to the *Msr1* promoter, thereby augmenting histone acetylation and subsequently enhancing *Msr1* transcriptional activity. This upregulation of MSR1 then contributes to elevated lipid uptake and consequently aggravating oxidative stress-dependent macrophage necroptosis. Additionally, FOXO3 also bound to the promoters of other regulated proteins associated with cell death, like *Tnfsf10*, *Cat,* and *Casp6*, and the binding was found to be abolished in *Pg*-stimulated macrophages. These genes may also participate in *Pg*-induced macrophage necroptosis.

Conventional treatment strategies for ASCVDs primarily center on lipid modulation and thromboprophylaxis.^62^ Despite notable therapeutic advancements, the persistent high global incidence and mortality of ASCVDs highlight the demand for innovative alternative therapeutic strategies. Given the crucial role of periodontal pathogens in atherosclerotic plaque instability and its underlying mechanisms involving macrophage lipid uptake, oxidative stress, and necroptosis, it merits further exploration whether suppressing macrophage lipid uptake and oxidative stress can exert therapeutic benefits on ASCVDs. Indeed, we observed that fucoidan and EUK134 treatment can significantly alleviate plaque instability. However, more specifically targeted pharmacological interventions for ASCVDs warrant further exploration.

## Materials and methods

### Human samples

In this study, total 33 atheromatous coronary arterial specimens were obtained from cardiac transplant patients diagnosed with severe ASCVDs at the Department of Cardiovascular Surgery, Union Hospital, Tongji Medical College, Huazhong University of Science and Technology (Wuhan, China), as described previously.^63^ This study (Approval No. 2021-0189) was conducted under the permission of the Ethics Committee of Union Hospital, Tongji Medical College, Huazhong University of Science and Technology (Wuhan, China). Briefly, recipient hearts were collected during surgery, and immediately immersed in ice-cold sterile saline. The left circumflex, left anterior descending, and right coronaries were excised and sectioned into small segments of 5 mm in length under sterile conditions. Coronary segments were fixed in 4% paraformaldehyde (PFA) or preserved at −80 °C for further detection. All patients and/or their immediate family members were informed of this experiment and signed the informed consent form. Detailed patient demographics and clinical features are tabulated in Table S1.

### Bacterial culture

*Pg* W83 was kindly provided by West China College of Stomatology, Sichuan University, China. The gingipains (Rgp & Kgp)-deficient *Pg* mutant strain KDP136 was kindly provided by Hospital of Stomatology, Sun Yat-sen University, China. *Pg* W83 and KDP136 were grown on Columbia blood agar plates (CP0160, Huankai Microbial, China) at 37 °C anaerobically for 5–7 days, and sub-cultured in hemin- and vitamin K (5 g/mL each)-supplemented brain heart infusion (BHI) medium (BD) under 37 °C anaerobic condition for 1 day.

### Rabbit model studies

Male New Zealand white rabbits (2.5–3.0 kg) were obtained from Wuhan Wonderjourney Biotechnology Co., Ltd. This experiment was conducted under the permission of the Institutional Animal Care and Use Committee (IACUC) of Tongji Medical College, Huazhong University of Science and Technology (IACUC number 4137).

To address the relationship between *Pg* and atherosclerotic plaque instability, rabbits were randomly assigned to three groups: untreated, periodontitis, and periodontitis with *Pg* infected groups (*n* = 6 in each group). In the periodontitis group, a 3-0 silk suture was ligated around bilateral mandibular second premolars. General anesthesia was administered via isoflurane inhalation (1.5–3%). In periodontitis with *Pg-*infected group, rabbits received periodontal ligature and additional topical applications of *Pg*-containing slurry to the ligature sites three times per week. The untreated group underwent no periodontal ligature. All experimental rabbits were individually housed with free water access and fed 0.5% cholesterol-supplemented high-fat diet for 16 weeks.

At the end of the experiment, rabbits were euthanized, and aortic arch specimens were harvested and fixed in 4% PFA for further analyses.

### Mouse model studies

6-week-old male C57BL/6 J *Apoe*^−/−^ mice were obtained from Beijing Vital River Laboratory Animal Technology Co., Ltd. These studies were carried out under the permission of the IACUC of Tongji Medical College, Huazhong University of Science and Technology (IACUC no. 3611). Because the protective effect of estrogen in ASCVDs in female, only male animals were involved in our experiments. To determine the appropriate group size, we reviewed the literature on atherosclerosis and noticed that six mice each group was acceptable.^64,65^ And by statistical analysis, we calculated the group sizes by using the formula (*n* = [(Z_1 − *α*/2_ + Z_1 − *β*_) (*κ* − 1)/(*δ*/*σ*)],^2^ *α* = 0.05 (two-tailed), *β* = 0.90, *n* indicates the sample size of each group, *κ* indicates the number of groups, *δ* indicates population difference of research significance, *σ* indicates population standard deviation) with the data of previous literature, and the result showed that five mice each group was statistically enough in our cases.^66^

Mice were housed under specific pathogen-free condition (23 ± 1 °C, 12 h light/dark cycle) under ad libitum access to both water and a western diet (D12079B, Jiangsu Xietong Pharmaceutical Bio-engineering Co., Ltd, China).

The inclusion criteria were all animals that survived all experimental procedures and completed the full experimental period. Animals failing to survive the study duration were excluded. Mice were subjected to the following experiments:

For analysis of the relationship between *Pg* infection and atherosclerotic plaque destabilization, *Apoe*^−/−^ mice were untreated, or intravenously injected with 10^7^ CFU *Pg* (suspended in 0.1 mL PBS) or PBS once per week for 20 weeks, 14 weeks, or 8 weeks. The untreated group underwent no injection.

To confirm the role of macrophage in *Pg*-induced atherosclerotic plaque destabilization, *Apoe*^−/−^ mice were administrated intraperitoneally with LC (0.2 mL, F70101C-A-2, FormuMax) for mononuclear phagocyte depletion, or vehicle once per 3 days, and intravenously injected with 10^7^ CFU *Pg* suspension or PBS once a week for 8 weeks. The first administration of LC was performed 24 h before *Pg* infection. The untreated group accepted no injection.

To investigate the effect of oxidative stress on *Pg*-promoted atherosclerotic plaque destabilization, EUK134 (10 mg/kg, S4261, Selleck) or DMSO (as control) was delivered via intraperitoneal injection to *Apoe*^−/−^ mice once per week, combined with or without intravenous injection with 10^7^ CFU *Pg* once per week for 8 weeks. The administration of EUK134 was performed 1 h before *Pg* infection. The untreated group underwent no injection.

To validate the role of gingipains in *Pg*-induced atherosclerotic plaque destabilization, *Apoe*^−/−^ mice were intravenously injected with or without 10^7^ CFU *Pg* or KDP136 suspensions, or PBS once a week for 8 weeks. The untreated group accepted no injection.

To identify the role of cell lipid uptake in *Pg*-caused atherosclerotic plaque destabilization, fucoidan (60 mg/kg, E0365, Selleck) or PBS was delivered to *Apoe*^−/−^ mice once per day, combined with or without *Pg* (10^7^ CFU in 0.1 mL PBS) infection through intravenous injection once a week for 8 weeks. The untreated group underwent no injection.

At the terminal endpoint of the study, mice were sacrificed, and the heart and aortic arch specimens were harvested, followed by fixation in 4% PFA, or preservation at −80 °C for further detection. The reagents used are provided in Supplementary Table 5.

### Histological staining

Serial 10-μm cross-sections were obtained from: (a) mouse aortic roots beginning at the first appearance of aortic valves, and (b) the same regions of rabbit aortic arches. Serial sections were allocated to consecutive slides. The whole sample was assessed at the same distance from the starting position. All parameters were quantified by multiple slides in each sample.

For analysis of atherosclerotic plaque instability, HE (S191003, Pinuofei), Oil red O (G1015-100ML, Servicebio), Masson (S191006, Pinuofei), and Prussian blue (P00129, Pinuofei) staining were applied. HE staining was employed to assess the area of the whole plaque and necrotic core. Lipid deposition was measured as the ratio of Oil Red O-positive area to total lesion area. Collagen content was expressed as the percentage of Masson-positive (blue) area within total lesion area. Fibrous cap thickness was assessed at its thinnest portion overlying most extensive necrotic core region. Prussian blue staining was employed to identify IPH. Quantification of stained area was assessed using NIH Image J software.

### Immunofluorescence

Cellular composition of atherosclerotic plaques was analyzed by immunofluorescent staining. Plaque macrophages were detected by co-staining of CD45 antibody (60287-1-Ig, Proteintech) and F4/80 (28463-1-AP, Proteintech), or CD68 (A23205, Abclonal). Smooth muscle cells were detected by ACTA2 (14395-1-AP, Proteintech). T cells were detected by CD3 (176175-1-AP, Proteintech). Neutrophils were detected by co-staining of CD45 antibody and Ly6G (65078-1-Ig, Proteintech). Cy3-conjugated secondary antibodies (SA00009-1, Proteintech), Alexa 488-conjugated secondary antibodies (SA00013-2, Proteintech), and FITC-conjugated secondary antibodies (AS019, Abclonal) were applied for detection. T cell levels were expressed as the percentages of CD3-positive cells in total intraplaque cells. Other data were described as proportions of fluorescent area in the whole lesion.

MSR1 (A2401, Abclonal), p-MLKL (Ab196436, Abcam), FOXO3 (10849-1-AP, Proteintech), NOX2 (19013-1-AP, Proteintech) expression, and gingipain (CSB-PA338957LA01PQP, Cusabio) existence in macrophages were assessed by immunofluorescence staining. 4,6-diamidino-2-phenylindole (DAPI, C1002, Beyotime) was applied to nuclei visualization. IgG of the primary antibody was utilized as immunostaining control to validate antibody specificity and to distinguish background from genuine target staining. Comprehensive antibody details are tabulated in Supplementary Table 2.

Images were acquired by Nikon A1-Si confocal microscope. The fluorescent area was quantified utilizing NIH Image J software.

### Fluorescence in situ hybridization (FISH)

Human arterial samples were sectioned into 5-μm-thick paraffin sections for FISH. FISH was conducted with a FITC-labeled oligonucleotide probe (5**′**-CAATACTCGTATCGCCCGTTATTC-3**′**, 5 mg/mL) targeting *Pg* ribosomal 16S rRNA sequences, followed by DAPI nuclear counterstaining.

### Transmission electron microscope (TEM)

Fresh segments of aortic arch tissues were carefully isolated and minced into 2 mm^3^ size and immediately fixed in electron microscope fixator (G1124-100ML, Servicebio) at 4 °C for primary fixation. After rinsing with 0.1 M phosphate buffer, the specimens underwent post 2-h fixation in phosphate buffer containing 1% osmium acid, followed by dehydration in graded ethanol (50–100%). Subsequently infiltrate and embed the samples in epoxy embedding medium (90529-77-4, SPI), followed by polymerization at 60 °C for 48 h. Ultrathin sections were made with a Leica UC7 ultramicrotome and stained with uranyl acetate and lead citrate (30 min each). Sections were imaged on a JEM-1400Flash TEM at 80 kV voltage.

### Determination of depletion efficiency of mononuclear phagocytes

At 72 h following intraperitoneal LC delivery, mice were anesthetized, and peripheral blood specimens (250 μL per mouse) were acquired via retro-orbital puncture into anticoagulated collection tubes. Erythrocytes were lysed through 5-minute incubation with ACK lysing buffer (Beyotime C3702) at a 1:3 sample-to-buffer ratio, and subsequently centrifuged at a low speed (500 × *g*, 5 min). After two PBS washes, leukocytes were immunolabeled using PE anti-mouse F4/80 (123109, BioLegend) and PerCP anti-mouse CD11b (101230, BioLegend). The samples were detected by flow cytometry.

### Cell culture

RAW264.7 murine macrophages (from China Center for Type Culture Collection) and THP-1 human monocytes (from American Type Culture Collection) were cultured in high-glucose DMEM and RPMI-1640 medium, respectively, both supplemented with 10% heat-inactivated fetal bovine serum. All cells were maintained at standard incubation conditions (37 °C, 5% CO_2_, 90–95% humidity).

### Cell apoptosis and necrosis assay

To detect intraplaque cell death, tissue sections were examined by TUNEL assay with in situ cell death detection kit (11684817910, Roche), complying with the instructions.

Apoptotic and necrotic cells in vitro were detected using Annexin V-FITC/PI Detection Kit (A211-01, Vazyme). The cells were collected, washed, and subsequently probed with PI and FITC-Annexin V for 15 min followed by flow cytometry analysis.

### Cell oil red O staining

Macrophages in 24-well plates were stimulated with *Pg* (MOI = 100) or PBS in the presence of ox-LDL (60 μg/mL, YB-002, Yiyuan biotechnology) for 24 h. Whereafter, cells underwent fixation with 4% PFA, and were immersed in Oil Red O solution (G1015-100ML, Servicebio) for half an hour. After extensive PBS washing, cells were observed under the microscope. The lipid accumulation level was represented as the proportion of Oil Red O-positive cells relative to total cells.

### Quantitative measurement of intracellular cholesterol

Macrophages in 12-well plates were stimulated with *Pg* (MOI = 100) or PBS in the presence of ox-LDL (60 μg/mL) for 24 h. Whereafter, macrophages were treated with pre-chilled RIPA lysis buffer and sonication. After centrifugation (12,000 × *g*, 10 min, 4 °C), supernatants were harvested for following detections. Protein concentrations were assessed using BCA assay (P0012S, Beyotime). The total cholesterol, cholesterol ester as well as free cholesterol concentrations were estimated using Total Cholesterol Assay Kit (BC1980, Solarbio) and normalized to total protein concentrations.

### Cholesterol efflux assay

Macrophages incubated with 1 μg/mL BODIPY-cholesterol (HY-125746, MedChemExpress) for 6 h. Then cellular monolayers were rinsed with PBS and underwent 24-h incubation with 5 μg/mL apo-AI (HY-P72833, MedChemExpress) and 20 μg/mL HDL (YB-003, Yiyuan biotechnology) as cholesterol receptors in serum-free medium, along with *Pg* (MOI = 100) or PBS co-stimulation. Fluorescence intensity (Ex/Em 505/515 nm) was determined in both supernatant and cell lysate. Cholesterol efflux level was calculated as: medium fluorescence/ (cellular fluorescence + medium fluorescence). The reagents used are provided in Supplementary Table 5.

### Lipid accumulation assay

After stimulation, macrophages in 12-well plates were stained with BODIPY 493/503 (10 μM, No. 25892, Cayman) for 30 min to detect ox-LDL uptake, or stained with Filipin (0.1 mg/mL, HY-N6716, MedChemExpress) for 30 min to detect free cholesterol content. After extensive PBS washing, cells were harvested, followed by flow cytometry analysis.

### Viral infection, RNA interference, and plasmid transfection

For *Msr1* gene silencing, macrophages were transduced with lentiviral vector (GV112, hU6-MCS-CMV-Puromycin, GENECHEM Biosciences) carrying *Msr1*-RNAi (NM_0011133269) at an MOI of 10. Following 24-h viral incubation in serum-free conditions, the old medium was substituted with complete growth medium containing puromycin (5 μg/mL) to select stably transduced cells.

For FOXO3 knockdown, macrophages were transfected with *Foxo3*-targeting siRNA oligonucleotides (GenePharma) using Lipo8000™ Transfection Reagent (C0533, Beyotime). Briefly, siRNA (0.1 μM) was mixed with Lipo8000 (6 μL) in serum-free medium (125 μL). After 15 min, the siRNA-Lipo8000 complex was added dropwise to cells. After 48-h incubation under standard culture conditions, cells were subjected to downstream assays.

The firefly luciferase reporter plasmids (PGL3-Basic, PGL3-*Msr1*, and their mutant/deletion variants) and the internal control Renilla luciferase plasmid pRL-TK were constructed by TsingKe Biological Technology (Wuhan, China). For transfection, cells were treated with a serum-free mixture containing the experimental reporter plasmids (500 ng), pRL-TK (125 ng), and Lipo8000 transfection reagent (1 μL). After 48-h incubation under standard culture conditions, cells were subjected to downstream assays.

### Protein extraction and western blot

Macrophages were sonicated ten times using a high-intensity ultrasonic processor in ice-cold RIPA buffer (P0013B, Beyotime) containing fresh inhibitors of protease (P1005, Beyotime) and phosphatase (P1081, Beyotime). Following centrifugation (12,000 rpm, 4 °C, 10 min), supernatant was mixed with loading buffer (P0015, Beyotime) and denatured (95 °C, 5 min). Samples were detected using the BCA assay to quantify the protein concentrations (P0012S, Beyotime) prior to separation by SDS-PAGE (G2177, Servicebio) and transfer to 0.45 μm PVDF membranes.

Following 1-h blocking using 3% skim milk, membranes underwent successive incubation with primary antibodies (4 °C, 12 h) and HRP-conjugated secondary antibodies (23 °C, 60 min). Blots were visualized using ECL system. Antibody information is exhibited in Supplementary Table 2.

### Detection of oxidative stress

For cultured macrophages, the ROS level was examined using the Total Oxygen Species Assay Kit (88-5930-74, Invitrogen). Briefly, following experimental treatments, cells were harvested and incubated with ROS Assay Stain for 60 min at 37 °C and 5% CO_2_. Fluorescence intensity was immediately measured by flow cytometry.

The ROS production within tissue sections from mouse aortic roots were measured using dihydroethidium (DHE, D7008, Sigma). Fresh segments of heart tissues were frozen in optimal cutting temperature compounds and sectioned into 10 μm-thick slices. Cryosections were incubated with 25 μg/mL DHE at 37 °C for 30 min. Cellular nuclei were probed using DAPI. Fluorescent images were taken by fluorescent microscopy (excitation 535 nm, emission 610 nm).

### Quantitative real-time PCR (qRT-PCR)

Total RNA was extracted employing Trizol reagent (R401-01, Vazyme) complying with recommended procedures. For reverse transcription, 1000 ng of extracted RNA served as the template for cDNA synthesis, which was performed utilizing the HiScript II Q RT SuperMix for qPCR kit (22107, TOBOLO). qRT-PCR was then performed utilizing AceQ Universal SYBR qPCR Master Mix (22204, TOBOLO) on a StepOne Plus real-time PCR platform (Applied Biosystems). Gene expression levels were normalized to β-actin and quantified via the 2^−ΔΔCt^ method. Primer information is exhibited in Supplementary Table 3.

### Silver staining

207.9 μmol/L recombinant human FOXO3 (CSB-EP008836HU1, Cusabio) was incubated with 2.1 μmol/L RgpA (ab225982, Abcam), RgpB (CSB-EP310587EYA, Cusabio) or Kgp (CSB- EP690409PQP1, Cusabio) (37 °C, 60 min), followed by SDS-PAGE. Subsequently, the gels were processed using the Fast Silver Stain Kit (P0017S, Beyotime), complying with recommended procedures. All relevant reagents are detailed in Supplementary Table 5.

### Chromatin immunoprecipitation (ChIP)

CHIP was performed utilizing the CHIP Kit (P2078, Beyotime) according to standardized protocols. Briefly, following formaldehyde crosslinking and cell lysis, DNA was sonicated into fragments less than 500 bp. Then, lysates were successively immunoprecipitated with 1 μg specific antibody (4 °C, 12 h), and protein A/G agarose beads (4 °C, 4 h). Antibody-chromatin complexes were washed sequentially with low salt, high salt, LiCl, and TE buffers, then eluted in elution buffer. Complexes were eluted and reversed in 200 mM NaCl (65 °C, 5 h). Purified DNA was obtained after RNase/proteinase K treatment and column purification. qRT-PCR was conducted using primers targeting the predicted FOXO3 binding sites on the *Msr1*, *MSR1*, *Tnfsf10*, *Cat*, and *Casp6* promoters. The DNA samples were detected for further 1% agarose gel electrophoresis. The CHIP-PCR primer information is exhibited in Supplementary Table 4.

### Luciferase reporter assay

Macrophages were seeded (10^5^ cells per well) in 24-well plates following transfection with either plasmids or an empty vector. After 48-h transfection, cells were treated with ox-LDL, combined with or without *Pg* or KDP136 stimulation for 24 h.

Following stimulation, cells were harvested and lysed for subsequent luciferase activity quantification. Firefly and Renilla luciferase activities were sequentially detected using the Dual-Luciferase Reporter Assay System (Promega). To assess relative *Msr1* promoter-driven transcriptional activity, the luminescence signal was normalized by calculating the Firefly luciferase (Fluc) to Renilla luciferase (Rluc) ratio.

### Co-immunoprecipitation (Co-IP)

Following stimulation, macrophages were lysed with Cell lysis buffer (P0013, Beyotime) containing fresh protease inhibitors (P1005, Beyotime) at 4 °C for 2 h. After centrifugation to remove debris, the lysates were successively incubated with specific primary antibodies (4 °C, 12 h) and with protein A agarose beads (PR40023, Proteintech) (4 °C, 4 h). Immunocomplexes-beads mixture was then washed with binding buffer for four times, and eluted in protein loading buffer (95 °C, 5 min). Samples were then subjected to western blotting analysis. Antibody information is exhibited in Supplementary Table 2.

### Proteomic analysis

Macrophages in 12-well plates were treated with *Pg* (MOI = 100) or PBS combined with ox-LDL (60 μg/mL) for 1 day. Whereafter, cells were collected and subjected to proteomic analysis. Protein was extracted as described before and underwent trypsin digestion. Then, peptides were subjected to TMT labeling using TMT reagents (Thermo Fisher Scientific) and analyzed through LC-MS/MS with EASY-nLC 1200 system, coupled to Q Exactive™ HF-X mass spectrometer using data-dependent acquisition (DDA) mode (350–1600 m/z at 60,000 resolution). Data were managed with MaxQuant search engine (v.1.6.15.0) against UniProt mouse FASTA database (Mus_musculus_10090_SP_20220107.fasta). FDR was adjusted to less than 1%. Differential proteins between *Pg* group and PBS control group with *P* value < 0.05 were regarded as statistically significant and identified as upregulated or downregulated proteins with a fold-change (*Pg*/PBS) > 1.15 or <0.87.

The raw mass spectrometry proteomics data have been deposited in the ProteomeXchange Consortium (https://proteomecentral.proteomexchange.org) via the iProX partner repository^67,68^ with the dataset identifier PXD048371.

### Statistical analysis

All statistical analyses were processed via GraphPad Prism 9.0. Shapiro–Wilk test was applied to examine data normality. For data with normal distribution, unpaired two-tailed Student’s *t*test was used for comparisons between two groups. One-way ANOVA, combined with Tukey post-hoc tests and repeated measure ANOVA, combined with Bonferroni post-hoc tests, were applied for multiple group comparisons. For non-normally distributed data, Mann–Whitney U test was used for comparisons between two groups, and Kruskal–Wallis test combined with Dunn’s multiple comparison tests was used for multiple group comparisons. For comparisons involving categorical variables, Fisher’s exact test was used. All results were described as mean ± standard error of the mean (SEM), or mean ± standard deviation (SD). n represented animal numbers or numbers of independent experiments in each experiment, as detailed in the figure legends. Values of *p* < 0.05 represented statistically significant difference.

## Supplementary information

Supplementary Materials

## Data Availability

All data may be obtained from the corresponding author upon reasonable request. The raw mass spectrometry proteomics data have been deposited to the ProteomeXchange Consortium (https://proteomecentral.proteomexchange.org) via the iProX partner repository^67,68^ with the dataset identifier PXD048371 at https://proteomecentral.proteomexchange.org/cgi/GetDataset?ID=PXD048371.
