# Supplementary material for: Porphyromonas gingivalis aggravates atherosclerotic plaque instability by promoting lipid-laden macrophage necroptosis
Source: Signal Transduct Target Ther. 2025 May 23;10:171. doi: 10.1038/s41392-025-02251-6 (PMC12098900; doi:10.1038/s41392-025-02251-6)
Supplement: Supplementary file 1 — Supplementary Materials [file 41392_2025_2251_MOESM1_ESM.docx]

Supplementary Materials for

*Porphyromonas gingivalis* aggravates atherosclerotic plaque instability by promoting lipid-laden macrophage necroptosis

Xiaofei Huang^1,2,3^, Mengru Xie^1,2,3^, Yixuan Wang^4,5^, Xiaofeng Lu ^1,2,3^, Feng Mei ^1,2,3^, Kaiwen Zhang^1,2,3^, Ying Yin^1,2,3^, Xinlong Yang^1,2^, Guangjin Chen^1,2,3^, Guangxia Feng^1,2,3^, Wencheng Song^1,2,3^, Nianguo Dong^4,5,^*, Xuliang Deng^6,^*, Songling Wang^7,8,^*, Lili Chen ^1,2,3,^*

Correspondence to: chenlili1030@hust.edu.cn

**This PDF file includes:**

Figures. S1 to S12

Tables S1 to S5

Figure. S1


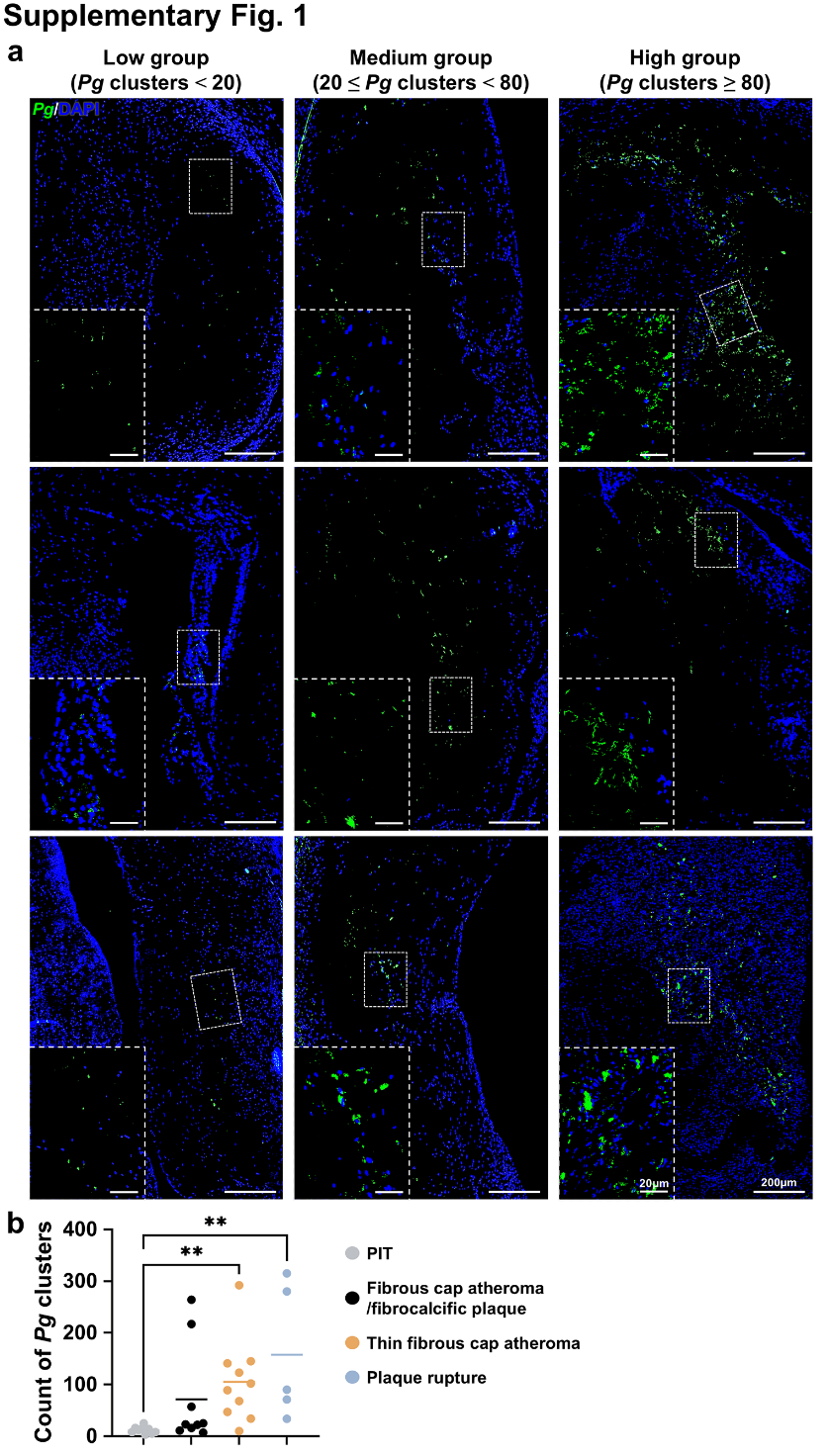


**Supplementary Figure 1.** *Pg* in atherosclerotic plaques of human coronary arteries. **a** Fluorescence in situ hybridization (FISH) detection of *Porphyromonas gingivalis* (*Pg*) in atherosclerotic plaques isolated from coronary vessels of atherosclerotic cardiovascular disease (ASCVD) patients. Group Low, the positive clusters of *Pg* ＜ 20; group Medium, 20 ≤ *Pg* clusters < 80; group High, *Pg* clusters ≥ 80. Nuclear DNA (blue) was counterstained with DAPI. Scale bar = 200 μm, and Scale bar = 20 μm for enlarged images. **b** Human coronary plaques were classified as pathological intimal thickening (PIT) (n = 9), fibrous cap atheroma or fibrocalcific plaque (n = 9), thin fibrous cap atheroma (n = 10), and ruptured plaque (n = 5), with the corresponding count of *Pg* clusters. Data were presented as mean. Data were analyzed by Kruskal-Wallis test. **P < 0.01.

Figure. S2.


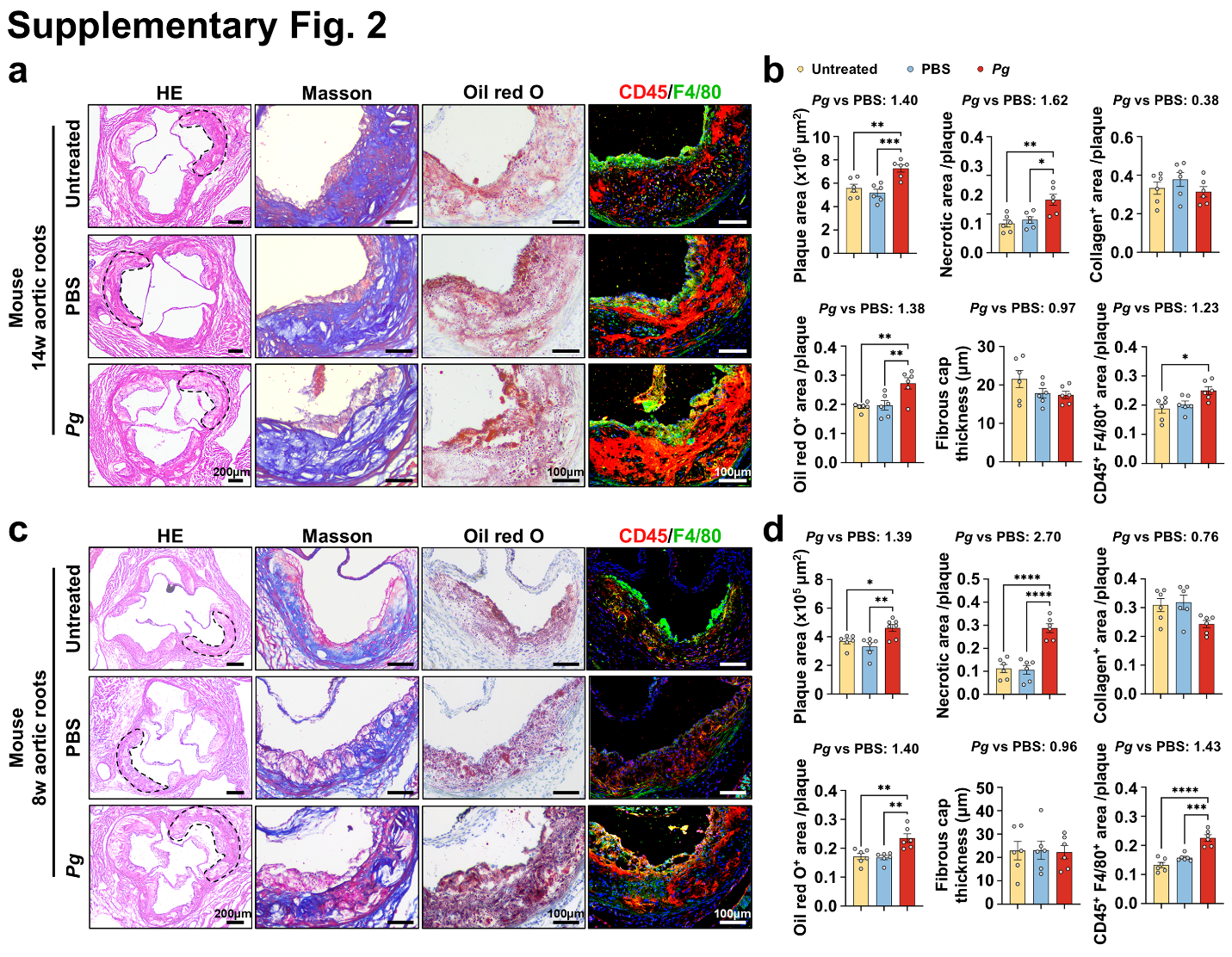


**Supplementary Figure 2.** *Pg* accelerates plaque vulnerability in earlier stages of atherosclerosis in mouse model. **a**, **c** H&E, Masson, Oil Red O staining, and CD45 and F4/80 co-staining (macrophage marker) of atherosclerotic plaques in aortic roots of *Apoe*^-/-^ mice untreated or treated with PBS or *Pg* for 14 weeks (**a**), and 8 weeks (**c**). Nuclear DNA (blue) was counterstained with DAPI. Scale bar = 200 μm in H&E, and 100 μm in the rest images. **b**, **d** Quantitative analyses of plaque size, necrotic area, collagen content, Oil Red O^+^ area, thickness of fibrous cap, CD45 and F4/80-positive areas of atherosclerotic plaques of *Apoe*^-/-^ mice modeled for 14 weeks (**b**), and 8 weeks (**d**). n = 6 per group. Data were presented as mean ± SEM. All data were analyzed by one-way ANOVA. ****P < 0.0001; ***P < 0.001; **P < 0.01; *P < 0.05.

Figure. S3.

**
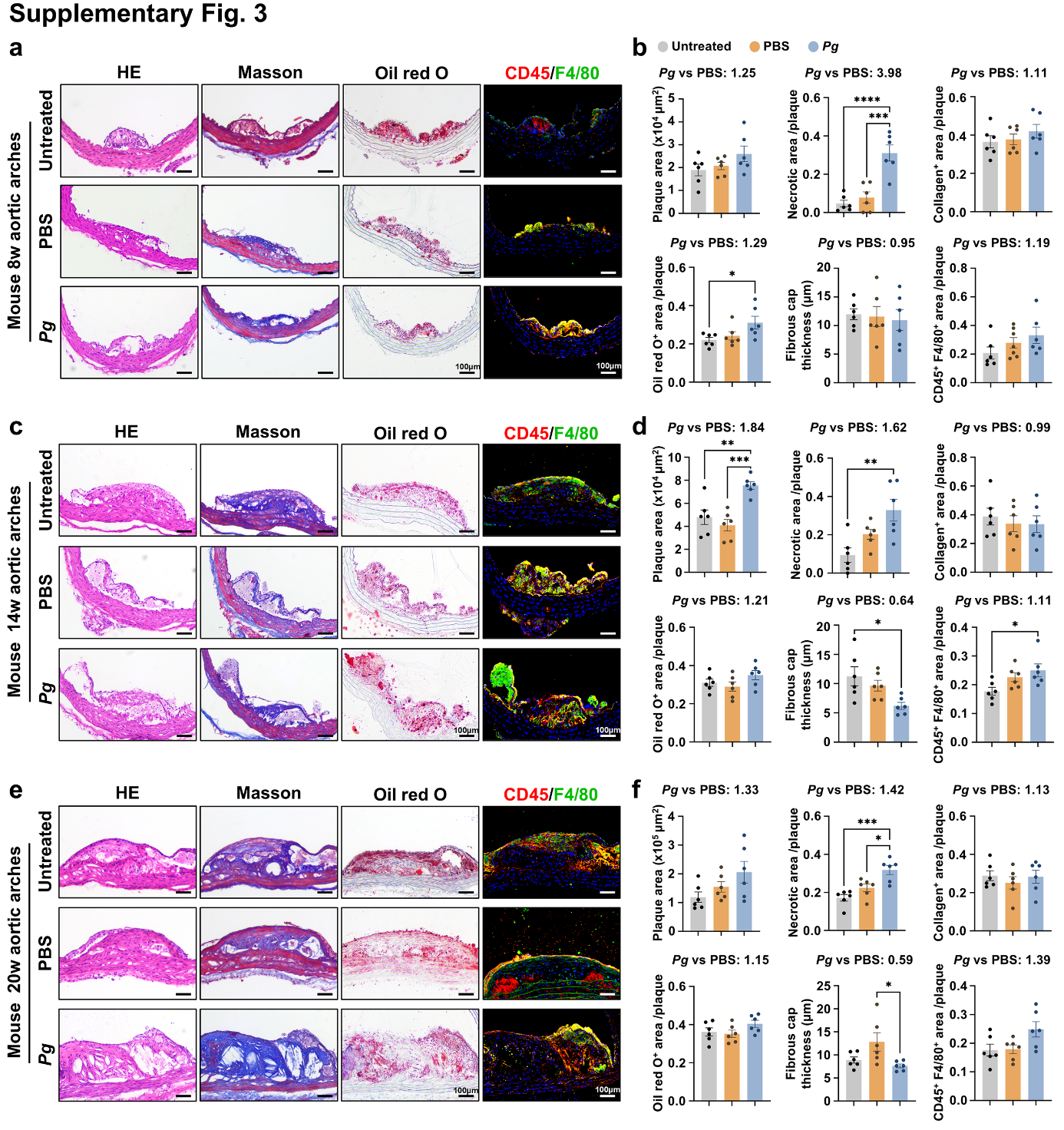
**

**Supplementary Figure 3.** *Pg* promote atherosclerotic plaque vulnerability in mouse aortic arches. **a**, **c** and **e** H&E, Masson, and Oil Red O staining, CD45 and F4/80 co-staining (macrophage marker) of atherosclerotic plaques in aortic arches of *Apoe*^-/-^ mice untreated or treated with PBS or *Pg* for 8 weeks (**a**), 14 weeks (**c**) and 20 weeks (**e**). Nuclear DNA (blue) was counterstained with DAPI. Scale bar = 100 μm. **b**, **d** and **f** Quantitative analyses of plaque size, necrotic area, collagen content, Oil Red O^+^ area, thickness of fibrous cap, CD45 and F4/80-positive areas of atherosclerotic plaques of *Apoe*^-/-^ mice modeled for 8 weeks (**b**), 14 weeks (**d**) and 20 weeks (**f**). n = 6 per group. Data were presented as mean ± SEM. All data were analyzed by one-way ANOVA. ****P < 0.0001; ***P < 0.001; **P < 0.01; *P < 0.05.

Figure. S4.

**
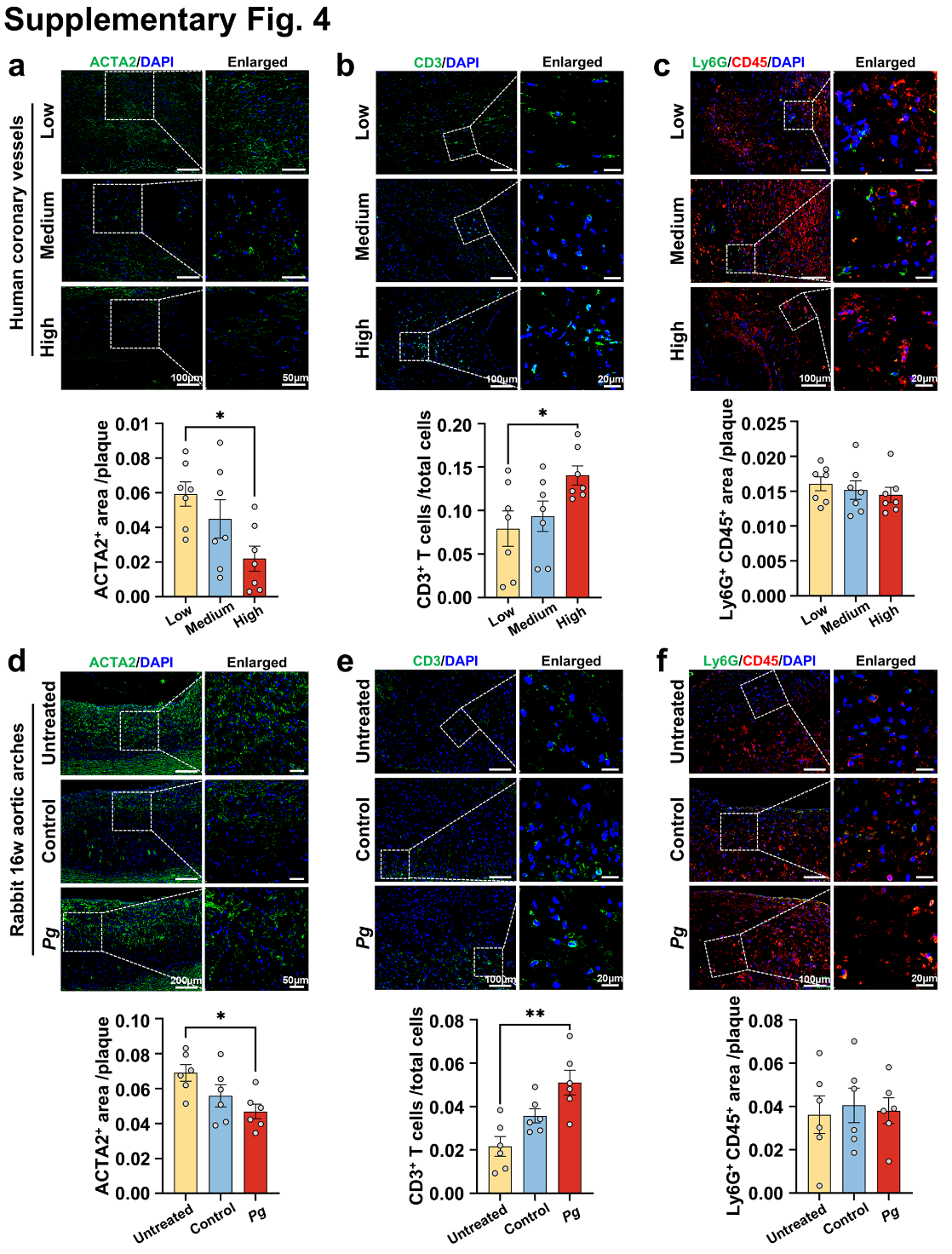
**

**Supplementary Figure 4.** Vascular smooth muscle cells, T cells and neutrophils in atherosclerotic plaques of human coronary arteries and rabbit aortic arches. **a** and **d** Detection and quantification of vascular smooth muscle cells (VSMCs) by ACTA2 immunohistochemical staining in plaques of human coronary vessels (**a**) (scale bar = 100 μm on the left, and 50 μm on the right), and rabbit aortic arches (**d**) (scale bar = 200 μm on the left, and 50 μm on the right.). **b** and **e** Detection and quantification of T cells by CD3 immunohistochemical staining in plaques of human coronary vessels (**b**), and rabbit aortic arches (**e**). Scale bar = 100 μm on the left, and 20 μm on the right. **c** and **f** Detection and quantification of neutrophils by CD45 and Ly6G co-staining in plaques of human coronary vessels (**c**), and rabbit aortic arches (**f**). Scale bar = 100 μm on the left and 20 μm on the right. Nuclear DNA (blue) was counterstained with DAPI. n = 7 per group in **a, b** and **c**; n = 6 per group in **d**, **e** and **f**. Data were presented as mean ± SEM. All data were analyzed by one-way ANOVA. **P < 0.01; *P < 0.05.

Figure. S5


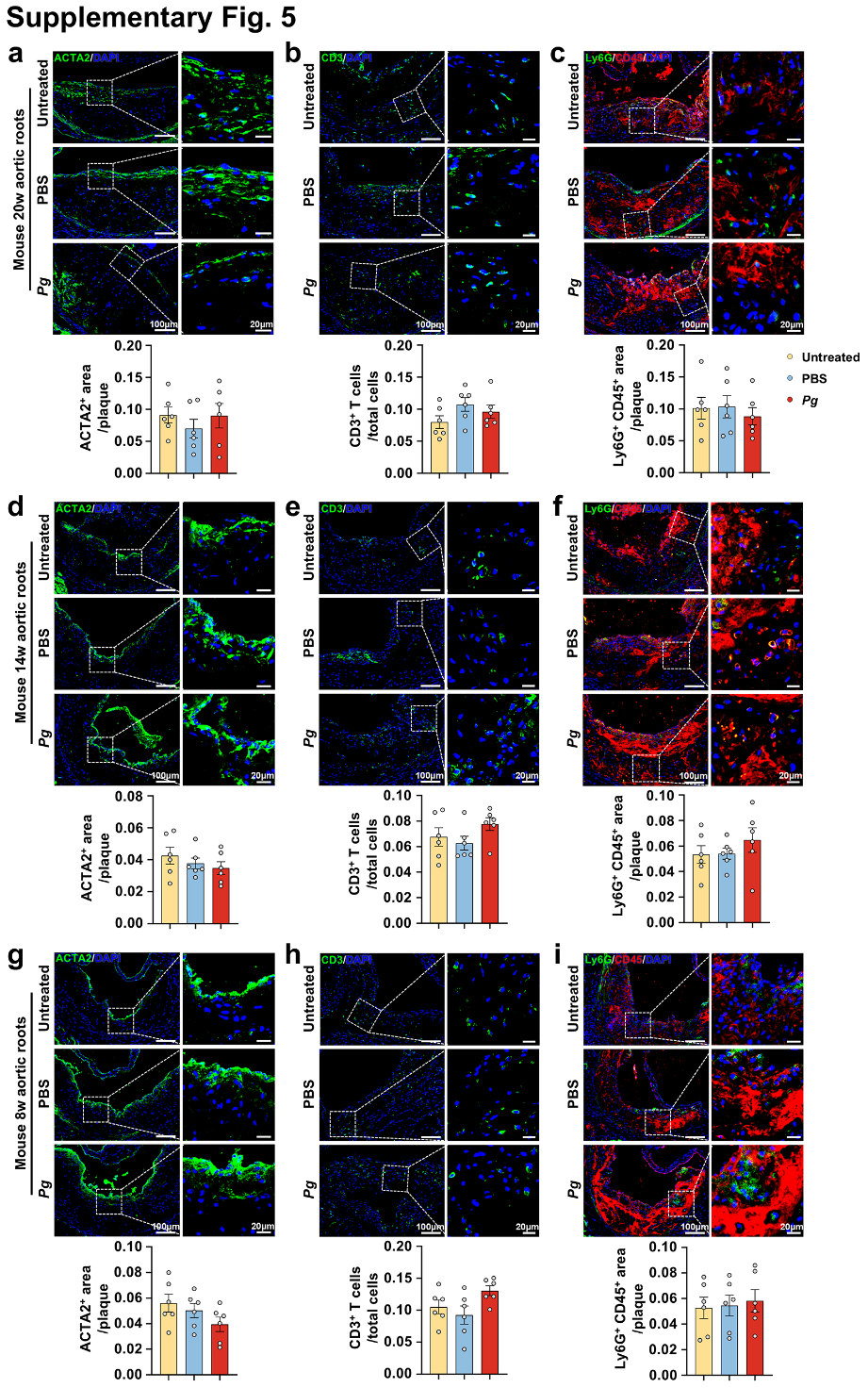


**Supplementary Figure 5.** Vascular smooth muscle cells, T cells and neutrophils in atherosclerotic plaques of mouse aortic roots. **a, d** and **g** Detection and quantification of VSMCs s by ACTA2 immunohistochemical staining in aortic root plaques of *Apoe*^-/-^ mice untreated or treated with PBS or *Pg* for 20 weeks (**a**), 14 weeks (**d**) and 8 weeks (**g**). Scale bar = 100 μm on the left, and 20 μm on the right. **b, e** and **h** Detection and quantification of T cells by CD3 staining in aortic root plaques of *Apoe*^-/-^ mice untreated or treated with PBS or *Pg* for 20 weeks (**b**), 14 weeks (**e**) and 8 weeks (**h**). Scale bar = 100 μm on the left, and 20 μm on the right. **c, f** and **i** Detection and quantification of neutrophils by CD45 and Ly6G co-staining in aortic root plaques of *Apoe*^-/-^ mice untreated or treated with PBS or *Pg* for 20 weeks (**c**), 14 weeks (**f**) and 8 weeks (**i**). Nuclear DNA (blue) was counterstained with DAPI. Scale bar = 100 μm on the left, and 20 μm on the right. n = 6 per group. Data were presented as mean ± SEM. All data were analyzed by one-way ANOVA.

Figure. S6.

**
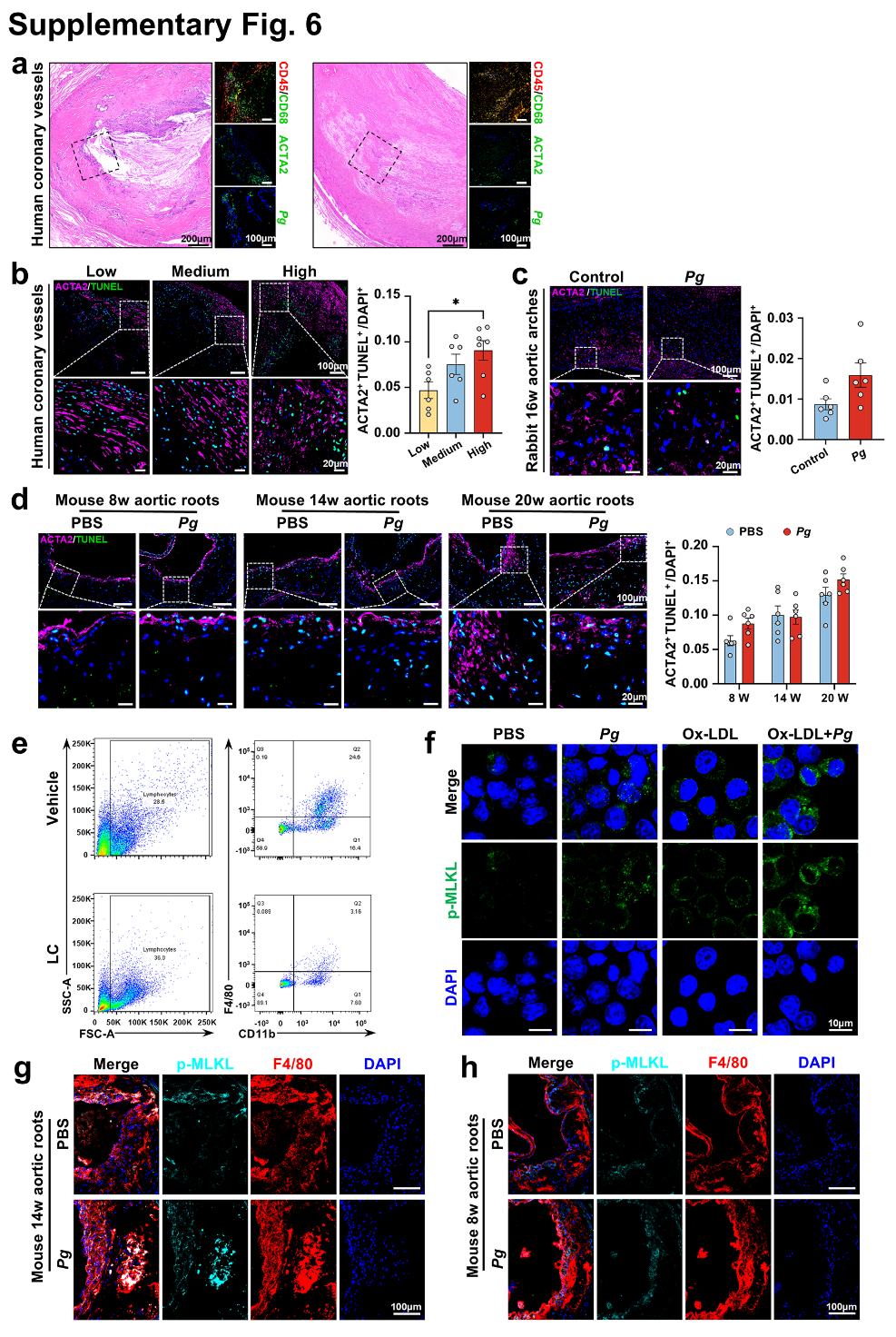
**

**Supplementary Figure 6.** *Pg* infection increased macrophage necroptosis in atherosclerotic plaques. **a** H&E, FISH, CD45 and CD68 (macrophage marker) co-staining and ACTA2 (VSMCs marker) staining of the necrotic core and extracellular lipid within human coronary plaques of high group. Scale bar = 200 μm in H&E, scale bar = 100 μm in the rest images. **b**, **c**, and **d** Detection and quantification of dying VSMCs probed by TUNEL and ACTA2 staining in the atherosclerotic plaques of human coronary arteries (**b**), rabbit aortic arches (**c**) and mouse aortic roots (**d**). n = 6-7 per group. Scale bar = 100 μm on top, and 20 μm on the bottom. **e** Flow cytometry analysis for F4/80 and CD11b-labled peripheral blood monocytes from *Apoe*^-/-^ mice with administration of vehicle or clodronate liposome (LC) for 8 weeks. **f** Immunohistochemical staining of p-MLKL in macrophages treated with ox-LDL (60 μg/ml) and/or *Pg* (MOI = 100) for 24 hours. Scale bar = 10 μm. **g** and **h** The co-localization of p-MLKL and F4/80-labeled macrophages in the aortic root plaques of *Apoe*^-/-^ mice infected with *Pg* for 14 weeks (**g**) and 8 weeks (**h**). Nuclear DNA (blue) was counterstained with DAPI. Scale bar = 100 μm. Data were presented as mean ± SEM. Statistical analyses were performed by one-way ANOVA (**b**), unpaired Student t test (**c**), or two-way ANOVA (**d**). *P < 0.05.

Figure. S7.

**
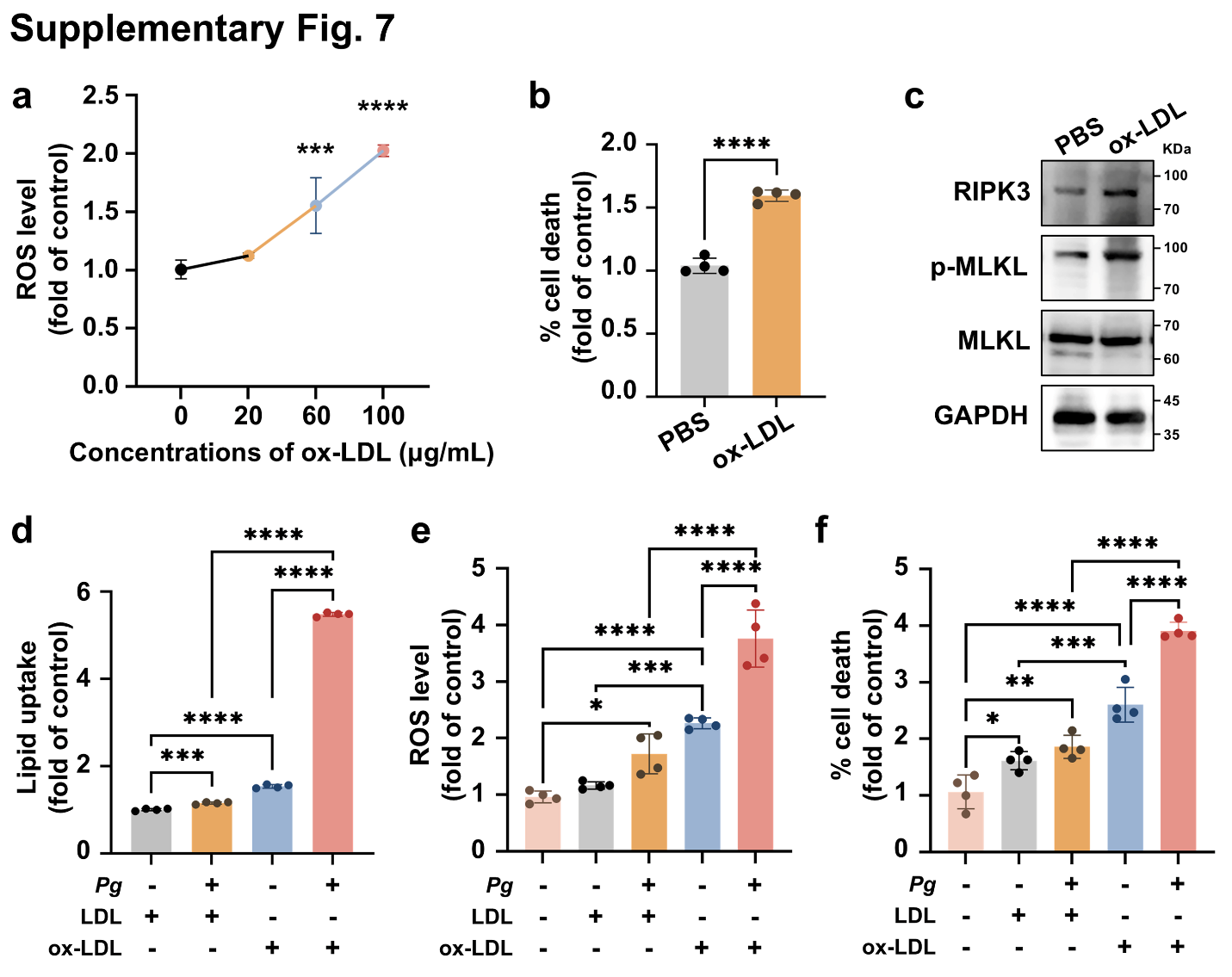
**

**Supplementary Figure 7.** Ox-LDL induces significant ROS production and increased necroptosis in macrophages. **a** Flow cytometry analyses of the ROS production in macrophages loaded with ox-LDL in different concentrations. n = 4 per group. **b** Flow cytometry analyses of the ratio of PI^+^ cells in macrophages stimulated with ox-LDL (60 μg/mL). n = 4 per group. **c** Western blot analyses of RIPK3, p-MLKL, MLKL, and GAPDH expression in macrophages administrated with ox-LDL (60 μg/mL) for 24 hours. GAPDH was used as the loading control. **d**, **e** and **f** Flow cytometry analyses of the lipid deposition (**d**), ROS production (**e**) and the ratio of PI^+^ cells (**f**) in LDL or ox-LDL (60 μg/mL)-treated macrophages challenged with or without *Pg* (MOI = 100). n = 4 per group. Data were presented as mean ± SD. Data were analyzed by one-way ANOVA (**a, d, e, f**) or unpaired two-tailed student t-test (**b**). ****P < 0.0001; ***P < 0.001; **P < 0.01; *P < 0.05.

Figure. S8.


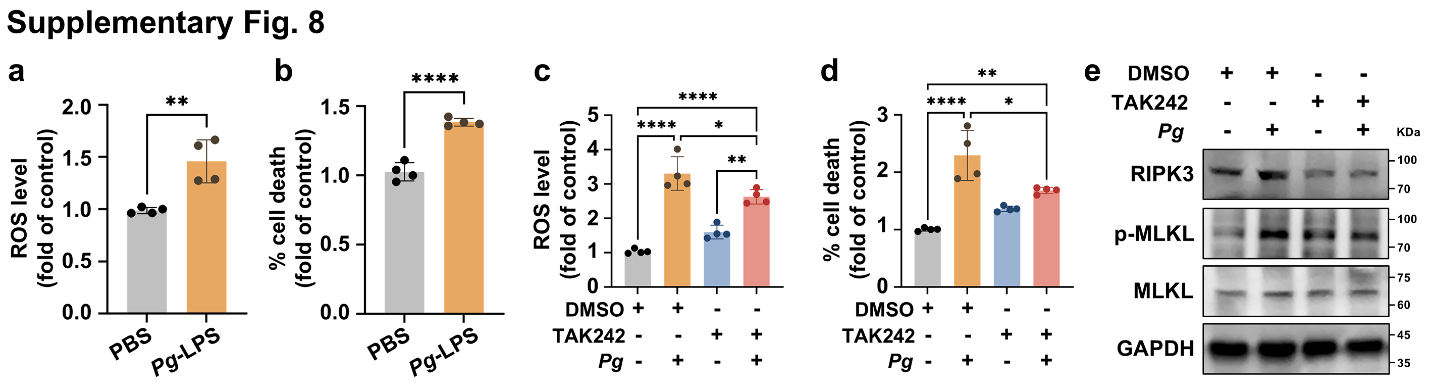


**Supplementary Figure 8.** The *Pg*-amplified ROS production and necroptosis do not solely depend on LPS. **a** and **b** Flow cytometry analyses of the ROS production (**a**) and the ratio of PI^+^ cells (**b**) in ox-LDL (60 μg/mL)-loaded macrophages challenged by *Pg*-LPS (1 μg/mL). n = 4 per group. **c** and **d** Flow cytometry analyses of the ROS production (**c**) and the ratio of PI^+^ cells (**d**) in ox-LDL (60 μg/mL)-loaded macrophages pre-treated with TAK242 (2 μM) and infected by *Pg* (MOI = 100). n = 4 per group. **e** Western blot analyses of RIPK3, p-MLKL, MLKL, and GAPDH expression in ox-LDL (60 μg/mL)-loaded macrophages treated with TAK242 (2 μM) and *Pg* (MOI = 100). GAPDH was used as the loading control. Data were presented as mean ± SD. Data were analyzed by unpaired two-tailed student t-test (**a**, **b**), or one-way ANOVA (**c**, **d**). ****P < 0.0001; **P < 0.01; *P < 0.05.

Figure. S9.


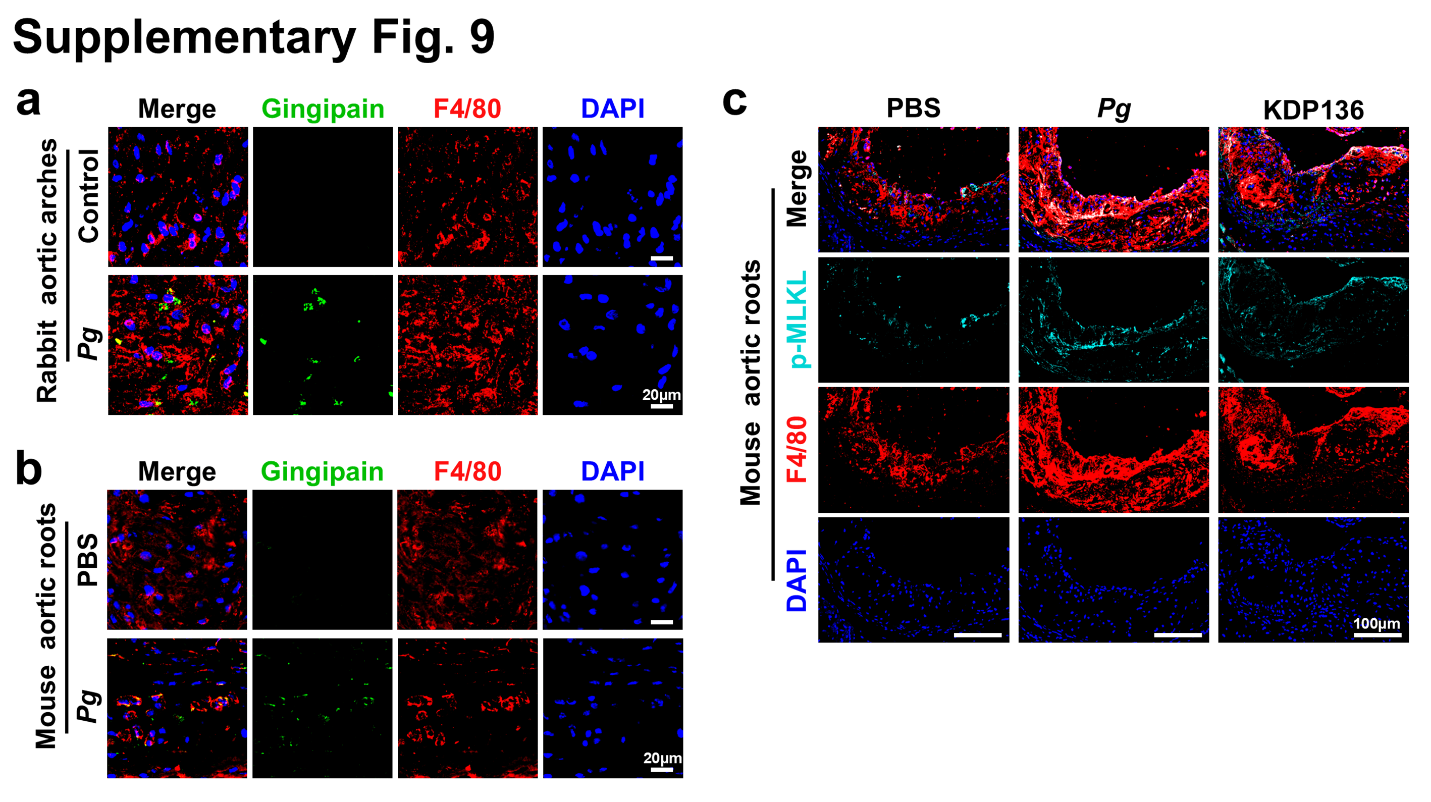


**Supplementary Figure 9.** Detection of gingipain existence and p-MLKL expression of macrophages in atherosclerotic plaques. **a** and **b** The co-localization of RgpA and F4/80-labeled macrophages in the aortic arch plaques of rabbits (**a**) and in the aortic root plaques of *Apoe*^-/-^mice (**b**). Scale bar = 20 μm. **c** The co-localization of p-MLKL and F4/80 labeled macrophages in the aortic root plaques of *Apoe*^-/-^mice infected with *Pg* or KDP136 for 8 weeks. Nuclear DNA (blue) was counterstained with DAPI. Scale bar = 100 μm.

Figure. S10.


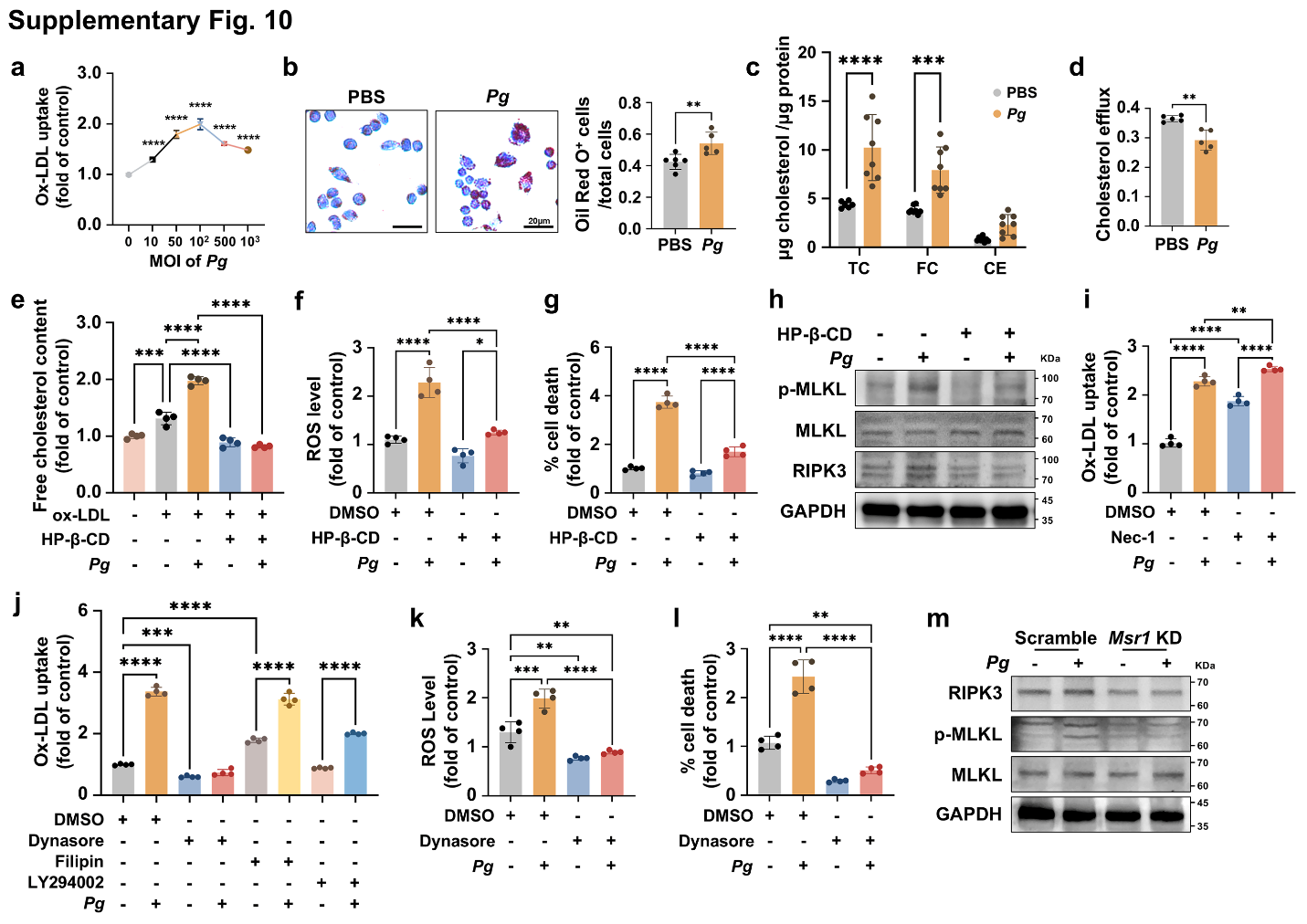


**Supplementary Figure 10.** *Pg*-promoted lipid uptake augmented ROS production and following necroptosis in macrophages. **a** Flow cytometry analyses of the ox-LDL uptake in macrophages infected with *Pg* of different MOIs. n = 4 per group. **b** Oil Red O staining of ox-LDL (60 μg/mL)-loaded macrophages inoculated with *Pg* (MOI = 100) for 24 hours. n = 5-6 per group. Scale bar = 20 μm. **c** Detection of cholesterol concentrations, including total cholesterol (TC), free cholesterol (FC) and cholesteryl ester (CE) in cell lysate of ox-LDL (60 μg/mL)-loaded macrophages inoculated with *Pg* (MOI = 100) for 24 hours. n = 8 per group. **d** Index of cholesterol efflux in ox-LDL (60 μg/mL)-loaded macrophages inoculated with *Pg* (MOI = 100). n = 5 per group. **e**, **f** and **g** Flow cytometry analyses of the FC content (**e**), ROS production (**f**), and the ratio of PI^+^ cells (**g**) in HP-β-CD (10 mM)-pretreated macrophages treated with or without ox-LDL (60 μg/mL) and/or *Pg* (MOI = 100). n = 4 per group. **h** Western blot analyses of RIPK3, p-MLKL, MLKL, and GAPDH expression in HP-β-CD (10 mM) pretreated macrophages loaded with ox-LDL (60 μg/mL) and *Pg* (MOI = 100). **i** Flow cytometry analyses of the ox-LDL uptake of macrophages under the administration of Nec-1 (10 μM) and/or *Pg* (MOI = 100). n = 4 per group. **j** Flow cytometry analyses of the ox-LDL uptake in macrophages pretreated with dynasore (10 mM), filipin (5 μM), or LY294002 (50 μM) and inoculated with/without *Pg* (MOI = 100). n = 4 per group. **k** and **l** Flow cytometry analyses of the ROS production (**k**) and the ratio of PI^+^ cells (**l**) in ox-LDL (60 μg/mL)-loaded macrophages pretreated with dynasore (10 mM) and inoculated with/without *Pg* (MOI = 100). n = 4 per group. **m** Western blot analyses of RIPK3, p-MLKL, MLKL, and GAPDH expression in *Msr1* knockdown macrophages preloaded with ox-LDL (60 μg/mL) and inoculated with *Pg* (MOI = 100). GAPDH was used as the loading control. Data were presented as mean ± SD. Data were analyzed by one-way ANOVA (**a, e-g**, **i-l**), unpaired two-tailed student t-test (**b, d**), or two-way ANOVA (**c**). ****P < 0.0001; ***P < 0.001; **P < 0.01; *P < 0.05.

Figure. S11.


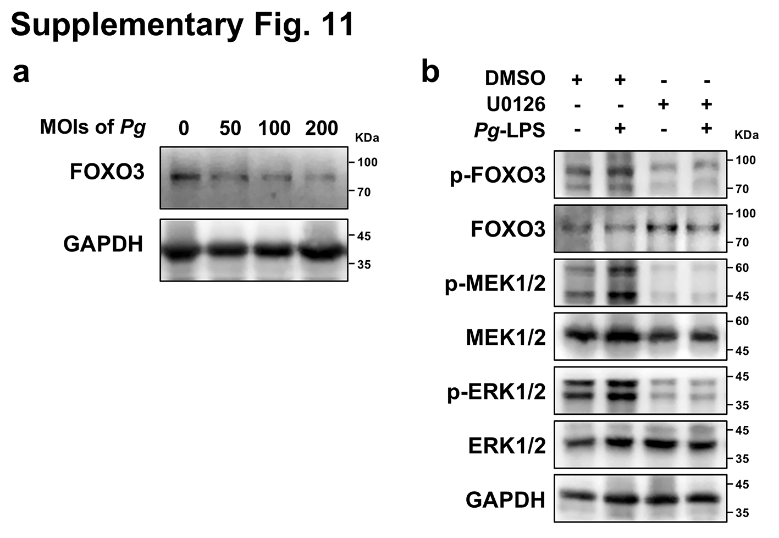


**Supplementary Figure 11. a** Western blot analyses of FOXO3 and GAPDH expression in ox-LDL (60 μg/mL)-loaded macrophages inoculated with *Pg* in different MOIs. **b** Western blot analyses of p-FOXO3, FOXO3, p-MEK1/2, MEK1/2, p-ERK1/2, ERK1/2 and GAPDH expression in U0126 (1 μM)-pretreated macrophages loaded with ox-LDL (60 μg/mL) and challenged by *Pg*-LPS (1 μg/mL). GAPDH was used as control.

Figure. S12.


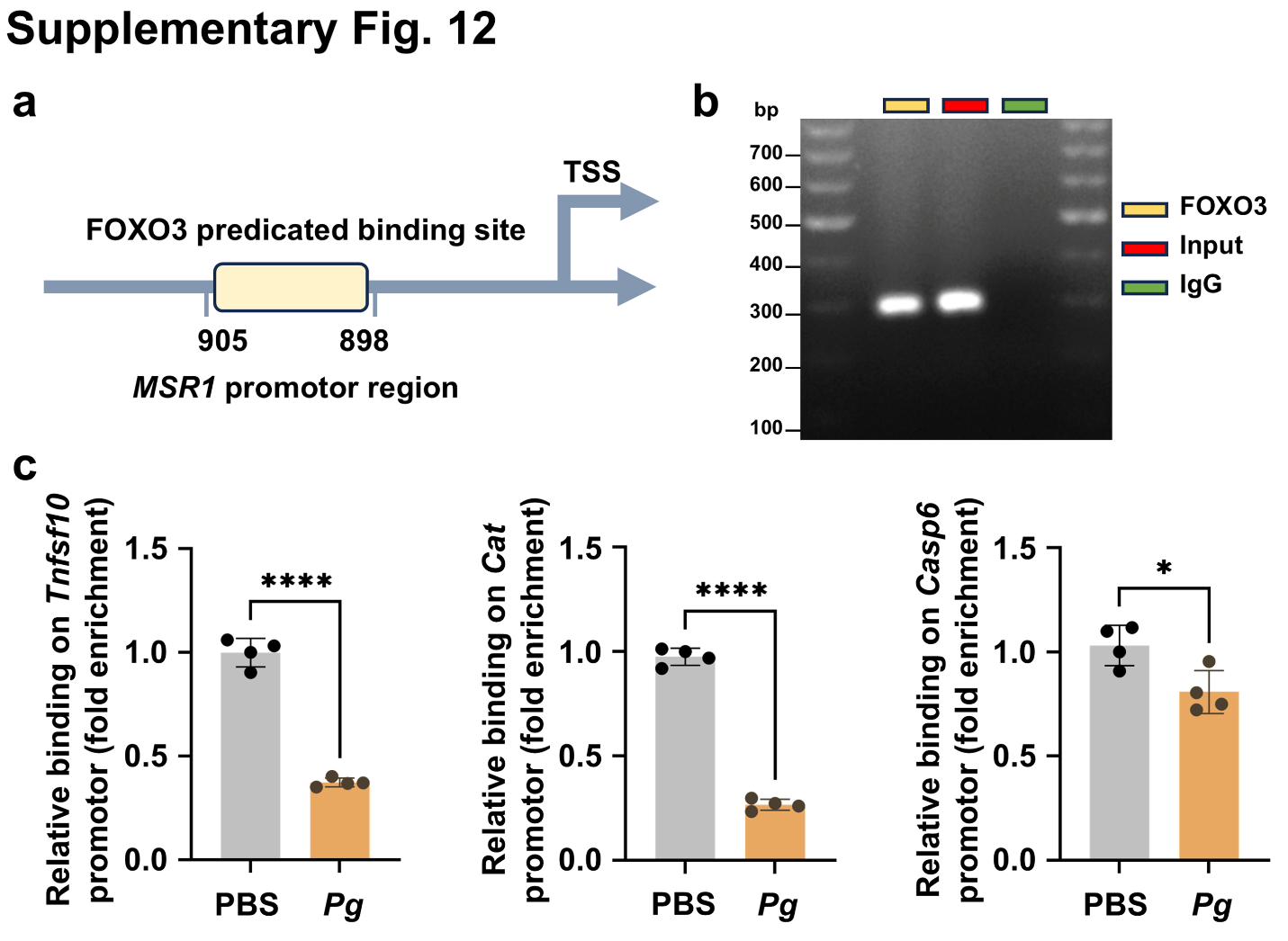


**Supplementary Figure 12. a** Predicted FOXO3 binding site on the human *MSR1* promoter according to JASPAR database. **b** CHIP followed by 1% agarose gel electrophoresis was performed to confirm the binding of FOXO3 on *MSR1* promotor in human THP-1 macrophages. An antibody for FOXO3 and an antibody for IgG as the negative control were used. **c** CHIP-PCR analysis of the binding of FOXO3 in *Tnfsf10*, *Cat* and *Casp6* promotors in ox-LDL (60 μg/mL) loaded-macrophages treated with/without *Pg* (MOI = 100). n = 4 per group. Data were presented as mean ± SD. Statistical analyses were performed by unpaired two-tailed student t-test. ****P < 0.0001; *P < 0.05.

Table S1.

Characteristics of patients.

| **Demographics** | **No. of patients (%)** |
| --- | --- |
| Total patients | 33 |
| Male / female | 31 (94) / 2 (6) |
| Age (years) | Mean 54 (range 28-67) |
| **Clinical data** | **No. of patients (%)** |
| NYHA functional class IV | 29 (87.88) |
| Major adverse cardiovascular events |  |
| Myocardial infarction | 8 (24.24) |
| Cerebral infarction | 5 (15.15) |
| Peripheral artery disease | 7 (21.21) |
| Treatments |  |
| Percutaneous coronary intervention | 12 (36.36) |
| Coronary artery bypass grafting | 3 (9.09) |
| Artificial heart implantation | 1 (3.03) |

Table S2.

Antibodies used in WB, IF, FC, CHIP, and Co-IP analysis.

| **Antibody** | **Origin** | **Catalog #** | **Assay** |
| --- | --- | --- | --- |
| CD45 | Proteintech | 60287-1-Ig | 1:200 IF |
| CD68 | ABclonal | A23205 | 1:200 IF |
| F4/80 | Proteintech | 28463-1-AP | 1:200 IF |
| ACTA2 | Proteintech | 14395-1-AP | 1:200 IF |
| CD3 | Proteintech | 176175-1-AP | 1:200 IF |
| Ly6G | Proteintech | 65078-1-Ig | 1:200 IF |
| MLKL | Proteintech | 66675-1-Ig | 1:1000 WB |
| Phospho-MLKL (S345) | Abcam | Ab196436 | 1:100 IF |
| Phospho-MLKL (S345) | Boster | P00535 | 1:1000 WB |
| RIPK3 | Proteintech | 17563-1-AP | 1:1000 WB |
| NOX2 | Proteintech | 19013-1-AP | 1:200 IF |
| MSR1 | ABclonal | A2401 | 1:1000 WB  1:200 IF |
| CD36 | ABclonal | A19016 | 1:1000 WB |
| OLR1 | ABclonal | A1639 | 1:1000 WB |
| RgpA | Cusabio | CSB-PA338957LA01PQP | 1:50 IF |
| FOXO3 | Proteintech | 10849-1-AP | 1:1000 WB  1:200 IF  1 μg/IP CHIP  4 μg/IP Co-IP |
| Phospho-FOXO3A (Ser315) | Proteintech | 28755-1-AP | 1:1000 WB |
| HDAC2 | Proteintech | 12922-3-AP | 1:1000 WB  4 μg/IP Co-IP |
| TPT1 | Proteintech | 10824-1-AP | 1:1000 WB |
| BNIP3 | Proteintech | 68091-1-Ig | 1:1000 WB |
| CD74 | ABclonal | A24487 | 1:1000 WB |
| HDAC4 | Proteintech | 66838-1-Ig | 1:1000 WB |
| PDCD4 | Proteintech | 12587-1-AP | 1:1000 WB |
| TAX1BP1 | Proteintech | 14424-1-AP | 1:1000 WB |
| Phospho-ERK1/2 (Thr202/Tyr204) | Proteintech | 28733-1-AP | 1:2000 WB  1:200 IF |
| ERK1/2 | Proteintech | 11257-1-AP | 1:1000 WB |
| Phospho-MEK1/MEK2 (S217/S221) | ABclonal | AP1349 | 1:1000 WB |
| MEK1/2 | Proteintech | 11049-1-AP | 1:2000 WB |
| Acetyl-Histone H4-K16 | ABclonal | A23091 | 1 μg/CHIP |
| Acetyl-Histone H3-K9 | ABclonal | A7255 | 1 μg/CHIP |
| GAPDH | Proteintech | 10494-1-AP | 1:5000 WB |
| HRP-conjugated Affinipure Goat Anti-Rabbit IgG (H+L) | Proteintech | SA00001-2 | 1:2000 WB |
| HRP-conjugated Affinipure Goat Anti-Mouse IgG (H+L) | Proteintech | SA00001-1 | 1:2000 WB |
| PE anti-mouse F4/80 | BioLegend | 123109 | 0.5 μg/10^6^ cells FC |
| PerCP anti-mouse CD11b | BioLegend | 101230 | 0.5 μg/10^6^ cells FC |
| Mouse IgG2b | Abcam | ab281590 | 1:200 IF |
| Rabbit IgG | Abcam | ab172730 | 1:200-1:100 IF  1 μg/IP CHIP  4 μg/IP Co-IP |
| Rat IgG | Beyotime | A7031 | 1:200 IF |
| Cy3–conjugated Affinipure Goat Anti-Mouse IgG (H+L) | Proteintech | SA00009-1 | 1:200 IF |
| Alexa 488-conjugated Goat Anti-Rabbit IgG (H+L) | Proteintech | SA00013-2 | 1:200 IF |
| FITC-conjugated Goat anti-Rat IgG (H+L) | ABclonal | AS019 | 1:200 IF |
| 4,6-diamidino-2-phenylindole (DAPI) | Beyotime | C1002 | 1:500 IF |

Table S3.

Primer sequences for qRT-PCR.

| **GENE** | **Forward** | **Backward** |
| --- | --- | --- |
| *Msr1* | GCACAATCTGTGATGATCGCT | CCCAGCATCTTCTGAATGTGAA |
| *Cd36* | ATGGGCTGTGATCGGAACTG | GTCTTCCCAATAAGCATGTCTCC |
| *Olr1* | CAAGATGAAGCCTGCGAATGA | ACCTGGCGTAATTGTGTCCAC |
| *Nos2* | GTTCTCAGCCCAACAATACAAGA | GTGGACGGGTCGATGTCAC |
| *Nox2* | TGTGGGAGACTGGACGGAG | CAGACTTGAGAATGGAGGC |
| *Sod1* | AACCAGTTGTGTTGTCAGGAC | CCACCATGTTTCTTAGAGTGAGG |
| *Sod2* | CAGACCTGCCTTACGACTATGG | CTCGGTGGCGTTGAGATTGTT |
| *Gpx1* | AGTCCACCGTGTATGCCTTCT | GAGACGCGACATTCTCAATGA |
| *Cox2* | TTCAACACACTCTATCACTGGC | AGAAGCGTTTGCGGTACTCAT |
| *Tax1bp1* | TCTGTTACGTCACCCATAAGGG | CCACCAACATATCGGAATTGCC |
| *Hdac4* | CTGCAAGTGGCCCCTACAG | CTGCTCATGTTGACGCTGGA |
| *Cd74* | AGTGCGACGAGAACGGTAAC | CGTTGGGGAACACACACCA |
| *Pdcd4* | CCACTGACCCTGACAATTTAAGC | TTTTCCGCAGTCGTCTTTTGG |
| *Bnip3* | GCAATGGCAATGGGAGCA | TGGTGTCTGGGAGCGAGGTG |
| *Tpt1* | CCATGACGAGCTGTTCTC | CACCCTCTGTTCTACTGACC |
| *Foxo3* | CATGCGCGTTCAGAATGAAGG | GACTGTCGTCTGCCGACTC |
| *β-actin* | GGCTGTATTCCCCTCCATCG | CCAGTTGGTAACAATGCCATGT |

Table S4.

Primer sequences for CHIP.

| **GENE** | **Forward** | **Backward** |
| --- | --- | --- |
| *Msr 1 Site#1* | ACCACGTGTCAGAAGGTACA | TCAGCCATCCCTGGCTAATAC |
| *Msr 1 Site#2* | CACCCCTGCCCACTTTTAGC | GCACCGACCATGAGAAGGCA |
| *MSR1* | ATCCATGCCTGAGATTCCTTCC | TCCTGTACCTTTATCCCTAGCC |
| *Tnfsf10* | GAGTCACAAACATTGGGCCAG | CCACACCACCACGAGTTTAC |
| *Catalase* | GCTTGGTAAATGATAATGAG | GGAGCGAAAGGTGGTGC |
| *Caspase 6* | CCTGTAGTGGGTT | CTTCCTTATTGTTTCA |

Table S5.

Reagents used in animal and cell experiments.

| **Reagents** | **Origin** | **Catalog #** | **Concentrations** |
| --- | --- | --- | --- |
| Ox-LDL | Yiyuan biotechnology | YB-002 | 60 μg/mL |
| DMSO | Sigma | 276855 | - |
| Clodronate liposomes | FormuMax | F70101C-A-2 | 0.2 mL/3 days |
| Z-VAD-FMK | MedChemExpress | HY-16658B | 5 μM |
| Necrostatin-1 | MedChemExpress | HY-15760 | 10 μM |
| Ferrostatin-1 | MedChemExpress | HY-100579 | 5 μM |
| VX765 | MedChemExpress | HY-13205 | 50 μM |
| EUK134 | Selleck | S4261 | 10 mg/kg/week;  10 μM |
| RgpA | Abcam | ab225982 | 1 μg/mL |
| RgpB | Cusabio | CSB-EP310587EYA | 1 μg/mL |
| Kgp | Cusabio | CSB- EP690409PQP1 | 1 μg/mL |
| *Pg*-LPS | Sigma | SMB00610 | 1 μg/mL |
| Fucoidan | Selleck | E0365 | 60 mg/kg/day；40 μg/mL |
| LDL | Yiyuan biotechnology | YB-001 | 60 μg/mL |
| Trichostatin A (TSA) | MedChemExpress | HY-15144 | 1 μM |
| TAK242 | MedChemExpress | HY-11109 | 2 μM |
| (2-Hydroxypropyl)-β-cyclodextrin (HP-β-CD) | MedChemExpress | HY-101103 | 10 mM |
| Dynasore | MedChemExpress | HY-15304 | 10 μM |
| Filipin | MedChemExpress | HY-N6716 | 0.1 mg/mL |
| LY294002 | MedChemExpress | HY-10108 | 50 μM |
| U0126 | Selleck | S1102 | 1 μM |
| Recombinant human FOXO3 | Cusabio | CSB-EP008836HU1 | 207.9 μmol/L |
| BODIPY-cholesterol | MedChemExpress | HY-125746 | 1 μg/mL |
| Apo-AI | MedChemExpress | HY-P72833 | 5 μg/mL |
| HDL | Yiyuan biotechnology | YB-003 | 20 μg/mL |
